# Signatures of landscape and captivity in the gut microbiota of Southern Hairy-nosed Wombats (*Lasiorhinus latifrons*)

**DOI:** 10.1186/s42523-020-00068-y

**Published:** 2021-01-06

**Authors:** Raphael Eisenhofer, Kristofer M. Helgen, David Taggart

**Affiliations:** 1 School of Biological Sciences, University of Adelaide, Adelaide, South Australia Australia; 2 Australian Research Council Centre for Australian Biodiversity and Heritage, University of Adelaide, Adelaide, South Australia Australia; 3 Australian Museum Research Institute, 1 William St, Sydney, New South Wales Australia; 4 Australian Research Council Centre for Australian Biodiversity and Heritage, University of New South Wales, Sydney, New South Wales Australia; 5 School of Animal and Veterinary Sciences (Waite), University of Adelaide, Adelaide, South Australia Australia; 6 FAUNA Research Alliance, PO Box 5092, Kahibah, NSW 2290 Australia

## Abstract

**Background:**

Herbivorous mammals co-opt microbes to derive energy and nutrients from diets that are recalcitrant to host enzymes. Recent research has found that captive management—an important conservation tool for many species—can alter the gut microbiota of mammals. Such changes could negatively impact the ability of herbivorous mammals to derive energy from their native diets, and ultimately reduce host fitness. To date, nothing is known of how captivity influences the gut microbiota of the Southern Hairy-nosed Wombat (SHNW), a large herbivorous marsupial that inhabits South Australia. Here, using 16S rRNA gene sequencing, we characterized the faecal microbiota of SHNWs in captivity and from three wild populations, two from degraded habitats and one from an intact native grass habitat.

**Results:**

We found that captive SHNWs had gut microbiota that were compositionally different and less diverse compared to wild SHNWs. There were major differences in gut microbiota community membership between captive and wild animals, both in statistically significant changes in relative abundance of microbes, and in the presence/absence of microbes. We also observed differences in microbial composition between wild populations, with the largest difference associated with native vs. degraded habitat.

**Conclusions:**

These results suggest that captivity has a major impact on the gut microbiota of SHNWs, and that different wild populations harbour distinct microbial compositions. Such findings warrant further work to determine what impacts these changes have on the fitness of SHNWs, and whether they could be manipulated to improve future management of the species.

**Supplementary Information:**

The online version contains supplementary material available at 10.1186/s42523-020-00068-y.

## Background

There is increasing recognition that host-associated communities of microorganisms (microbiota) play key roles in animal health and should be a considered factor in wildlife management practices [1]. The gut microbiota has been demonstrated to influence host health through interactions with the immune system, behaviour, digestion, and essential nutrient synthesis (reviewed in [2–4]). For instance, herbivorous mammals can harbor microbes that detoxify plant defence compounds [5], increasing their dietary niche breadth. Additionally, it has long been understood that the fermentation of plant compounds by the gut microbiota, for example within the complex compartmentalized digestive systems of foregut fermenters like ruminant mammals, can make substantial contributions to the daily energy requirements of herbivores. It has been estimated that short-chain fatty acids (SCFA) produced by the gut microbiota of Southern Hairy-nosed Wombats (SHNWs), a hindgut fermenter, account for > 60% of the daily energy requirement of the host [6]. Loss or disruptions of these host-associated microbiota and functions could therefore have major implications for the health and fitness of animals.

Captive breeding is an important wildlife management tool for many species. However, captivity can drastically modify the natural mammalian gut microbiota through various factors including changes in diet and antibiotic treatment [7–10] (reviewed in [1]). Diet is known to have a major influence on the gut microbiota of mammals [11]. Sonnenburg et al. [12] demonstrated that a change in diet can shift gut microbiota diversity and composition in laboratory mice (*Mus musculus*) and that, while these shifts were reversible in a single generation by dietary changes, they could not be restored by dietary intervention alone after multiple generations. Such microbial ‘extinctions’ were only reversible by reintroduction of both the missing microbes and diet. Martínez-Mota et al. [13] showed that white-throated woodrats (*Neotoma albigula*) brought into captivity and fed their natural diet retained more of their native microbiota when compared to animals fed an artificial diet. Determining which mammals experience effects of captivity on their gut microbiota is an important first step in developing management practices to retain native gut microbial diversity.

Animals of the same species living in geographically different populations can also have distinct gut microbiota compositions, which may be locally adaptive. Black howler monkeys (*Alouatta pigra*) were found to have habitat-specific gut microbiota signatures [14]. Monkeys living in degraded forests harboured lower gut microbial diversity and fewer microbes capable of producing butyrate, a SCFA energy source for the host. Similar trends were found in another study on two populations of Udzungwa red colobus monkeys (*Procolobus gordonorum*) [15]. The gut microbiota of animals living within the same population can also differ substantially. Koalas (*Phascolarctos cinereus*) from the same population were found to have distinct gut microbiota signatures associated with the consumption of different *Eucalyptus* species [16], and emerging evidence suggests that the gut microbiota of koalas can influence diet selection [17]. Identifying inter- and intra-population gut microbiota signatures could therefore yield important information from which to make wildlife management decisions, such as coordination/matching of individuals and locations to maximise translocation success.

To date, little is known about the gut microbiota of the Southern Hairy-nosed Wombat (SHNW) (*Lasiorhinus latifrons*). The SHNW is a large, sedentary, semi-fossorial marsupial herbivore, found in semiarid grassland habitats across southern South Australia, west of the Murray River to the south-eastern corner of Western Australia [18–20]. It is a grazer and a hindgut fermenter, with microbially-facilitated digestion occurring especially in an expansive colon [6, 21, 22]. Animals are subjected to annual extremes in water and nutrient availability, with limited access to water or food during summer and autumn months. Feed becomes prevalent after rainfall, which typically occurs in winter and spring, unless the region is in drought [18, 23, 24]. The species displays significant physiological and behavioural adaptations for water and energy conservation in this harsh environment, including a low basal metabolic rate, ever-growing cheek teeth well-adapted to mechanically reducing food into small particulates, long gut retention times, production of dry faecal pellets, and a relatively inactive, burrowing lifestyle [22–27]. The home range of this species is very small (2–4 ha) for an animal of their size and centered around their preferred warrens [28]. In intact habitats, animals feed mostly on native perennial grasses - *Austrostipa* spp., forbs and chenopods (saltbush - *Atriplex, Enchylaena* and *Rhagodia* spp., and bluebush - *Maireana* spp.) and it is recognized as an important ecosystem engineer [24, 29]. When native vegetation is unavailable, as in degraded habitats, a variety of introduced weed species are eaten [24]. The species is also of conservation concern, with a formerly broad geographic range in southern Australia fragmented by agriculture, livestock production, and other land-use changes, and is currently listed by the IUCN as “Near-threatened.” It is also closely related to the critically endangered northern hairy-nosed wombat (*Lasiorhinus krefftii* [30]). Nothing is known about whether habitat type influences the gut microbiota of SHNWs, or whether different populations of SHNWs have distinct gut microbiota.

To better understand whether factors such as captivity or habitat type influence the gut microbiota of SHNWs, we collected and characterised the microbial composition of fresh faecal samples from one captive and three wild populations in South Australia (two of which represent degraded, and one intact habitat). We hypothesised that 1) captivity results in both the reduction of diversity and a substantial change in gut microbial composition, and 2) different wild populations harbour distinct gut microbial compositions.

## Methods

### Study sites and habitat

#### Captive

Captive SHNWs accessed for this study were held by an experienced wildlife carer in the Adelaide Hills, approximately 10 km south-west of Adelaide, South Australia. These were rescue animals brought in from across the state, with most being hand-reared from a young age. The animals were housed in separate, custom-built enclosures containing burrows, and fed Barastoc Complete Performer (primarily cooked and flaked barley and lupins, lucerne and cereal chaff). The diet was bolstered in the wintertime with sunflower, oats, carrots, and sweet potato. Animals had access to water ad-lib.

#### Degraded habitat - Kooloola Station and Brookfield Conservation Park

**Fig. 1:**
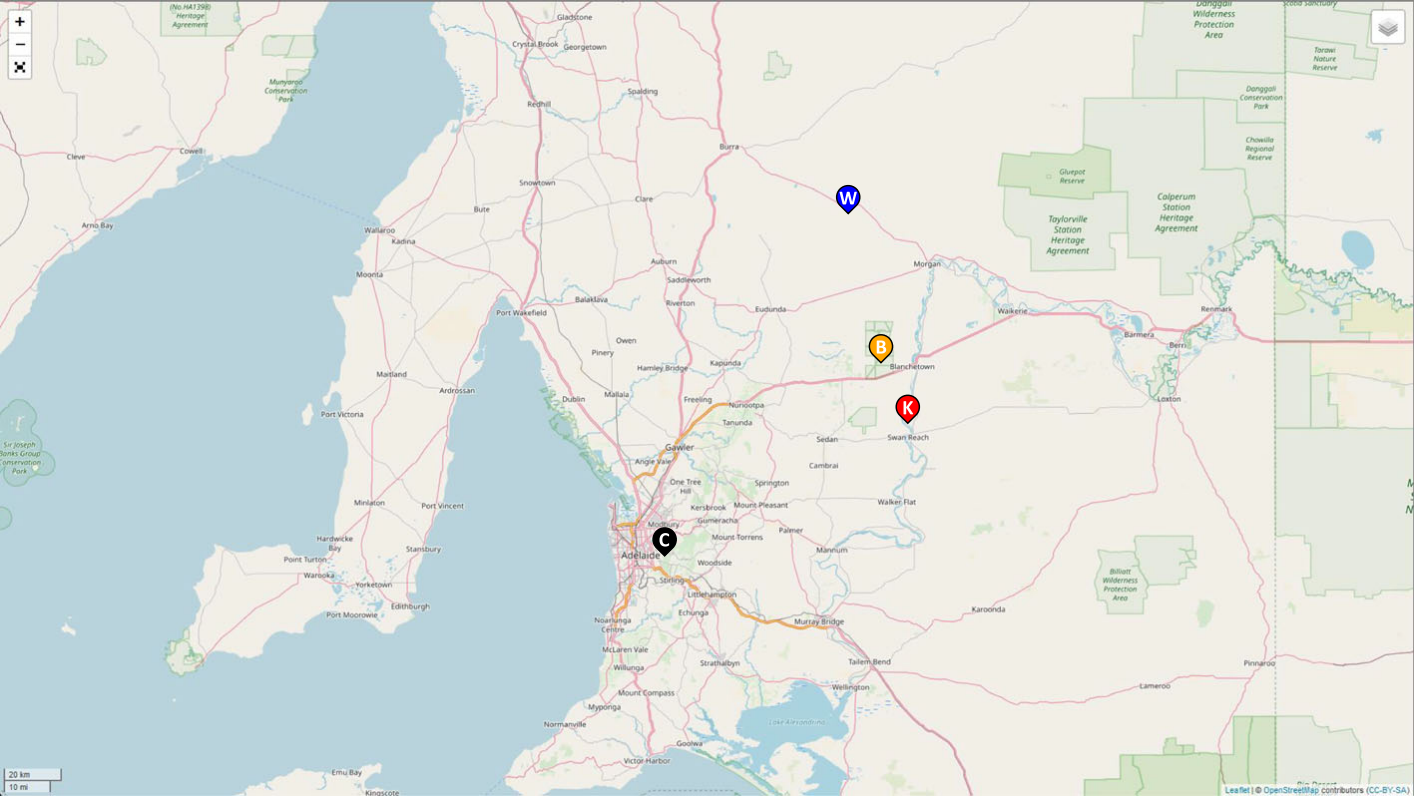
Map of study sites in South Australia. C = captive population (*n* = 21), K = Kooloola Station, degraded habitat (*n* = 21), B = Brookfield Conservation Park, degraded habitat (*n* = 17), and W = Wonga Station, intact habitat (*n* = 23)

Wild SHNWs from degraded habitat were located at Kooloola Station and Brookfield Conservation Park (Fig. 1). Both sites were historic sheep grazing properties and are located approximately 2.5 h drive north east of Adelaide, in South Australia’s Murraylands region. The annual rainfall in this area is ~ 270 mm (Bureau of Meteorology - BOM, 2018). The area consists of remnant mallee eucalypt woodland with a chenopod and grassland understory composed of a variety of introduced weeds, native grasses and chenopods. The diet of wombats in this area is made up largely of introduced weed species including thread iris (*Moraea setifolia*), wards weed (*Carrichtera annua*) and burr medic (*Medicago minima*), along with remnant chenopods and a few native grass species [31]. Except immediately after rain, no free water is available at these sites.

#### Intact habitat - Wonga Station

Wild SHNWs from intact habitat were located at Wonga Station, a sheep grazing property of ~ 130,000 acres, located about 3.5 h drive north east of Adelaide, in South Australia’s Murraylands region (Fig. 1). This area receives ~ 290 mm of rainfall annually (BOM, 2018). The vegetation on this property is predominantly composed of intact, native grassland habitat interspersed with remnant mallee eucalypt woodland. Active weed management is undertaken at this site. Native grasses and forbs, the dominant component of wombat diet in this region, flourish at this site and include many *Stipia*, *Hyalosperma*, *Silene, Rytidiosperma* and *Sida* spp., and a wide variety of Chenopodiaceae (saltbush - *Atriplex, Enchylaena* and *Rhagodia* spp., and bluebush - *Maireana* spp.) among others [31]. Except immediately after rain, no free water is available at this site.

### Sample collection

Once faeces have been deposited by an animal, the microbes inside can continue to grow and distort the true representation of the microbial community as it was inside the host [32]. To counteract this potentially confounding variable, faecal samples are best collected fresh and preserved. Freezing is a commonly used preservative method, but is difficult to use in a fieldwork context. Instead, we opted for preservation of faecal samples by immersion in 95% ethanol, which has been demonstrated to reliably preserve faecal microbial community composition at room temperature [32]. We collected samples in the evening/early morning when SHNWs are most active, offering the best opportunity to collect fresh faecal samples.

The entrance and soil mound area around SHNW warrens were searched for fresh faecal pellets. Old pellets and fresh pellets were qualitatively distinguished by gently squeezing with a freshly gloved hand. Once found, fresh pellets were placed on a piece of aluminium foil and cut in half using the cutting edge of a spatula. The pellet cores were extracted using the opposite end of the spatula and placed in 2 mL plastic screw-top tubes prefilled with 1.5 mL of 95% ethanol, before being shaken vigorously to ensure mixing of sample with ethanol. Gloves were changed between samples, and spatulas were decontaminated using 5% bleach (sodium hypochlorite) followed by an ethanol wash.

Samples from Kooloola Station (*n* = 21 from 11 warrens) were collected in the evening of 13 March 2019; Brookfield samples (*n* = 17 from 8 warrens) were collected in the morning of 14 March 2019 (mean overnight temperature of 7.2 °C; Eudunda weather station, Bureau of Meteorology). Samples from Wonga Station (*n* = 23 from 11 warrens) were collected overnight and into the morning of 19 June 2019 (mean overnight temperature of 1 °C; Eudunda weather station, Bureau of Meteorology). For detailed maps of the sample sites, see https://github.com/EisenRa/2020_SHNW_Faecal_16S/tree/main/Site_maps. Scat samples from captive animals (*n* = 21) were collected between 13 May 2019 and 5 June 2019, with some individuals being sampled up to three different time points (multiple samples from the same individual were not pooled, but processed separately and sample names suffixed with: a, b, c). A soil sample from each wild study site was also collected for analysis.

### DNA extraction

All DNA extractions were performed in freshly decontaminated Perspex hoods in a pre-PCR laboratory to prevent contamination with amplicons [33]. DNA was extracted using the QIAGEN DNeasy PowerSoil kit (formerly MO BIO PowerSoil) according to the manufacturer’s protocol. To reduce contamination, all buffers and tubes needed for the various steps were aliquoted prior to opening any sample tubes. Faecal samples were centrifuged at 10,000 g for 5 min before pouring the ethanol off. Because SHNW faeces are very dry, only ~ 150 mg from each sample was used. To minimise batch effects samples in extraction groups were randomized, and to account for laboratory related contamination extraction blank controls from each extraction group was included and carried through to DNA sequencing [33].

### Amplicon library preparation, quantification, and DNA sequencing

All samples were PCR-amplified and uniquely barcoded for High-Throughput Sequencing (HTS) using primers targeting the V4 region of the bacterial 16S rRNA gene [34]. We used forward primer: 515F (AATGATACGGCGACCACCGAGATCTACACTATGGTAATTGTG-TGCCAGCMGCCGCGGTAA) and barcoded reverse primer 806R (CAAGCAGAAGACGGCAT-ACGAGATnnnnnnnnnnnnAGTCAGTCAGCCGGACTACHVGGGTW TCTAAT) – the 12 n’s represent unique barcode sequences. The PCR reactions were prepared in a pre-PCR laboratory in a 5% bleached-cleaned and UV irradiated hood. Single reactions [35] of 2.5 μL X10 HiFi buffer, 0.1 μL Platinum™ Taq DNA Polymerase (ThermoFisher), 19.2 μL dH2O, 0.2 μL 100 mM dNTP mix, 0.5 μL each of 10 μM forward and reverse primer and 1 μL DNA. DNA was amplified using an initial denaturation at 94 °C for 3 min, followed by 35 cycles of denaturation at 94 °C for 45 s, annealing at 50 °C for 1 min, elongation at 68 °C for 90 s, with final adenylation for 10 min at 68 °C, in line with the Earth Microbiome Protocol [36].

Gel electrophoresis was carried out for each PCR reaction on a 3.5% agarose gel to ensure the samples contained library constructs of the desired length (~ 390 bp). For each sample, 1 μL amplified DNA was mixed into 199 μL Qubit® working solution (diluted Qubit® dsDNA HS Reagent 1:200 in Qubit® dsDNA HS Buffer) and quantified using a Qubit® 2.0 Fluorometer. Samples were pooled equimolar and cleaned using AxyPrep™ (Axygen) following the manufacturer’s instructions. Because negative controls contained little DNA, they were pooled separately and spiked into the final pool at a flat volume [33]. The final pool was quantified and quality checked using an Agilent TapeStation. DNA sequencing was performed on an Illumina MiSeq (v2, 2 × 150 bp) at SAHMRI (South Australian Health and Medical Research Institute).

### Data processing and statistical analyses

DNA sequencing data were processed and analysed using QIIME2 v2020.2 [37]. An interactive Jupyter notebook containing all the QIIME2 code used is available (https://github.com/EisenRa/2020_SHNW_Faecal_16S/blob/main/SHNW_Gut_16S_2019.ipynb). For captive animals that had multiple collections we randomly selected one sample for further downstream processing (see the Jupyter notebook for details). Forward reads (R1) were imported into the QIIME2 and denoised into amplicon sequence variants (ASVs) using the deblur [38] plugin with a trim length of 150 bp. Representative sequences were assigned taxonomy using the QIIME2 feature-classifier plugin (naive bayesian approach) on the pre-trained SILVA [39] 132 V4 region classifier [40]. A phylogenetic tree was created using the SATé-enabled phylogenetic placement (SEPP) technique [41] within the fragment-insertion QIIME2 plugin. Alpha diversity (Observed OTUs and Faith’s Phylogenetic diversity [42]) and Beta diversity (weighted [43] and unweighted [44] UniFrac metrics) were calculated using the QIIME2 diversity plugin with a rarefaction depth of 36,346 sequences per sample (see https://github.com/EisenRa/2020_SHNW_Faecal_16S/blob/main/SHNW_Gut_16S_2019.ipynb for the rarefaction curve). Tests for differentially abundant microbes between populations were performed using ANCOM [45]. SINA (SILVA Incremental Aligner) [46] was used to search specific ASVs against the SILVA 138 database. We used SourceTracker2 (https://github.com/biota/sourcetracker2) [47] to assess whether microbes from the soil were a source for faecal samples. PCoA, Alpha diversity, and Venn diagrams were constructed using phyloseq [48] and ggplot2 [49] in R Studio [50], see the Rmarkdown file for details (https://github.com/EisenRa/2020_SHNW_Faecal_16S/blob/main/Figures.rmd). Maps were generated using Geoplaner (www.geoplaner.com) with data from OpenStreetMaps (wiki.openstreetmap.org).

### Removal of outlier samples

We observed 7 samples collected from the 4 Eastern-most warrens from Brookfield Conservation Park that appeared to be outliers in our dataset. PCoA of unweighted UniFrac distances clearly separated these samples from other wild and captive samples (SI Figure 1). Additionally, the diversity in these outlier samples was higher than other Brookfield samples (mean of 1256 observed ASVs vs. 828). The taxonomic composition of these outlier samples was also substantially different to other samples (SI Figure 2). An interactive view at different taxonomic levels can be observed by dragging SI File 2 into the https://view.qiime2.org/ webpage. The most stark difference between these outlier samples and the other samples in the dataset is the loss of *Spirochaetes*, which was present in all other wild samples with a mean relative abundance of 11.6% and captive samples at 5.1%. Additionally, these samples did not have any *Izimaplasmataceae* (average 5.4% in the other wild samples). A possible cause for these differences could be taphonomy—perhaps these faecal samples were older than they seemed, which could have resulted in a shift of observed community composition. Alternatively, there could be true biological differences between these samples that we were unable to determine due to our non-invasive sampling technique (host health or disease, etc.). Due to these samples being so different to other wild samples (greater than between population differences or captive vs. wild) we chose to exclude these outlier samples from subsequent analyses.

### Frequency-based filtering to mitigate cross-sample contamination

Samples undergoing DNA extraction and library preparation in the same batch can be affected by cross-sample contamination, whereby cells (from DNA extraction) or DNA (from DNA extraction and/or library preparation) can cross from a sample to another [33, 51]. Such cross-sample contamination can therefore yield false-positive detection of microbes within samples, with samples of higher microbial biomass being more likely to spillover [51]. Because we randomized sample order within DNA extractions and library preparations (to reduce batch effects), there is a possibility of spillover between faecal samples from different populations. This would confound attempts at identifying ASVs that are unique to a particular population (i.e. Figs. 3b, 5). To try and mitigate the effect of cross-sample contamination for population-specific ASV analysis, we applied a conservative frequency-based filtering approach, whereby population-specific ASV tables were filtered to remove ASVs with a relative abundance of < 0.00015%). This threshold was based on the frequency of the most abundant ASV in the dataset (3c44df3672100a011a334b67eea24366), which was found in all wild samples with a total frequency of 278,742 reads (5.8% of total reads in wild samples). In contrast, this ASV was only found in the captive-only table with a total frequency of 131 reads (0.0001% of the captive table abundance). Assuming that the most abundant ASV would result in the greatest number spillover events, setting a threshold based on this ASV (0.00015%) would account for the spillover of less abundant ASVs. See the interactive Jupyter notebook for more details and the exact code ran (https://github.com/EisenRa/2020_SHNW_Faecal_16S/blob/main/SHNW_Gut_16S_2019.ipynb). The issue of cross-samples contamination in microbiome studies clearly deserves further research.

## Results

DNA sequencing of the 97 samples resulted in 13,545,820 reads (mean of 139,647), which were denoised using deblur into 8483 amplicon sequence variants (ASVs).

### Captivity influences SHNW gut microbiota diversity and composition

**Fig. 2:**
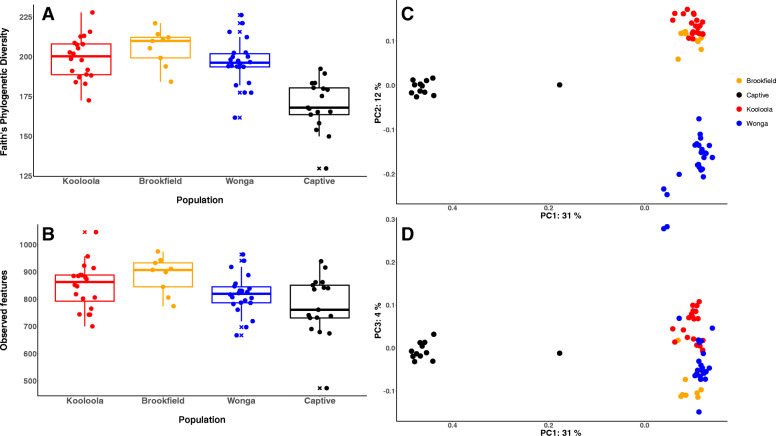
Microbial diversity and composition of SHNW faecal samples from different populations: Kooloola and Brookfield (degraded habitat), Wonga (intact habitat), and captive. **a** Captive individuals had significantly lower phylogenetic diversity of microbes compared to wild individuals, and **b** significantly lower microbial richness (except vs. Wonga). Horizontal lines in the boxes represent the median values; the lower and upper bound of boxes represent 25th and 75th percentiles, respectively. Outliers are marked with a cross. **c/d** Principal coordinates analysis (PCOA) of unweighted UniFrac distances show clear separation of microbial compositions between captive and wild individuals across PC1. **c** shows PC1 vs PC2 and **d** shows PC1 vs. PC3. Each point represents a SHNW faecal sample, coloured by population, and the distances between points represents microbial composition differences (i.e. points closer together have more similar microbial composition)

Microbial diversity was higher in wombat scats collected from wild populations compared to those sourced from captivity (Fig. 2a & b). All three wild populations had statistically significantly higher microbial phylogenetic diversity versus the captive population (Fig. 2a; Kruskal-Wallis tests of Faith’s phylogenetic diversity *p*-values < 0.001 SI Table 1). This finding was mirrored with microbial richness, with the exception of captive versus Wonga (Fig. 2b; Kruskal-Wallis tests of richness *p-*values < 0.05 SI Table 2). Analysis of microbial composition via principal coordinates analysis (PCoA) of unweighted UniFrac distances showed clustering of samples by captivity status (Fig. 2c & d). Captive samples were separated from wild samples across the first principal coordinate, which explained 31% of the variation, and these differences in microbial composition were statistically significant (PERMANOVA of unweighted UniFrac distances *p*-value = 0.001, pseudo-F 26.7). This result was also found when using the weighted (by abundance) Unifrac metric (SI Figure 3; PERMANOVA of weighted UniFrac distances *p*-value = 0.001, pseudo-F 19.4).

**Fig. 3:**
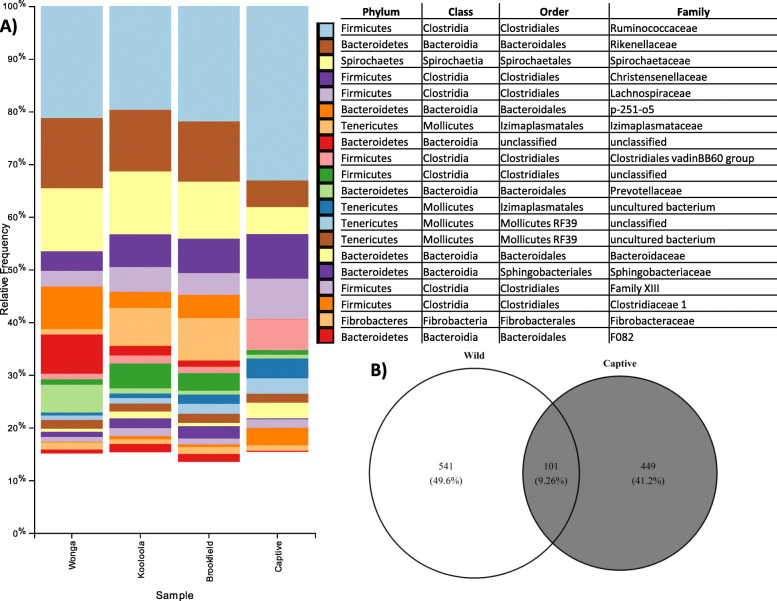
Family-level microbial taxonomic bar charts of the 20 most abundant families collapsed by population, ordered from most abundant family (top) to least (bottom) (**a**). Only the top 20 most abundant families are displayed for clarity. Venn diagram of ASVs found uniquely or shared between captive and wild individuals (**b**). The size and overlap of regions is weighted by the number of ASVs with the percentage of total ASVs listed for each region. Wonga (intact habitat), Kooloola and Brookfield (degraded habitat)

The microbial phyla with the greatest relative abundance in SHNW faecal samples were *Firmicutes* (39.5 and 64.4% relative abundance for wild and captive, respectively), *Bacteroidetes* (29.7% wild, 13.3% captive), *Tenericutes* (12.1% wild, 10.3% captive), and *Spirochaetes* (11.6% wild, 5.1% captive) (SI File 1). The *Firmicutes*:*Bacteroidetes* ratio was substantially higher in captive animals (mean 4.8:1) compared to wild (Kooloola & Brookfield = 1.7:1, Wonga = 0.8:1). Twenty five microbial families accounted for ~ 90% of the faecal microbiota, with the three most abundant families being *Ruminococcaceae* (20.9% wild, 33.1% captive), *Spirochaetaceae* (11.5% wild, 5.1% captive), *Rikenellaceae* (12.2% wild, 5.1% captive), and *Christensenellaceae* (5.5% wild, 8.5% captive) (Fig. 3 and SI File 1). Interestingly, some families were only detected in wild animals; *Izimaplasmataceae* (5.4% in wild), *p-251-o5* (5.3% in wild), and an unclassified *Bacteroidia* family (3.5%). We used ANCOM to statistically test whether there were any families of microbes that were differentially abundant between captive and wild individuals. Twenty-one families were found to be significantly differentially abundant between captive and wild individuals (SI File 2).

At the sequence-level, we found 181 ASVs that were significantly differentially abundant between captive and wild individuals (SI File 3). We next sought to determine whether there were ASVs only detected in captive or wild SHNWs. After filtering feature tables to conservatively account for cross-contamination between samples (see methods), the total number of ASVs in the captive samples went from 2618 (1,292,572 total sequences) to 550 (1,107,746), and 3096 (5,026,870) to 642 (4,729,528) for wild samples. From these ASVs, 449 were found only in captive SHNWs, accounting for 73.7% of the total abundance, with 541 ASVs being found only in wild SHNWs (71.3% abundance). One hundred and one ASVs were shared between the captive and wild SHNWs, accounting for 26.3 and 28.7% of their faecal microbiota abundance, respectively (Fig. 3b). To determine the distribution of these ASVs across the samples, we calculated the core microbiota (i.e. ASVs found in at least N% of samples) for both between and within captive and wild SHNWs. Six ASVs were found in all samples (0.5% of total ASVs), and 31 were found in at least 90% of all samples (2.8% of total ASVs) (Table 1). Thirty-seven ASVs were found in all captive samples (6.7% of total captive ASVs), and 105 were found in at least 90% of captive samples (19% of total captive ASVs). Forty-nine ASVs were found in all wild samples (7.6% of total wild ASVs), and 193 ASVs were found in at least 90% of wild samples (30% of total wild ASVs).

**Table 1:** Core ASV statistics between and within different SHNW populations

Population	Captive + Wild	Captive	Wild
**# sequences**	5,837,274	1,107,746	4,729,528
**# ASVs**	1091	550	642
**# 100% core ASVs**	6	37	49
**% of total ASVs**	0.5%	6.7%	7.6%
**# sequences from 100% core ASVs**	103,657	98,989	953,577
**% abundance**	1.8%	8.9%	20.2%
**# 90% core ASVs**	31	105	193
**% of total ASVs**	2.8%	19%	30%
**# sequences from 90% core ASVs**	1,073,221	268,413	2,983,540
**% abundance**	18.4%	24.2%	63.1%

We next used SINA (SILVA Incremental Aligner) to contextualize the most abundant core ASVs (SI Table 3). The most abundant 100 and 90% core ASVs to all samples were classified as *UCG-005* and *Treponema*, respectively, with a 97.3 and 95.3% nucleotide identity to the references. The most abundant 100 and 90% core ASV in the captive samples was the same *UCG-005* ASV. The most abundant 100% core ASV in the wild samples was classified to *Treponema*, with a 90.7% nucleotide identity to the reference. Finally, the most abundant 90% core ASV in the wild samples was also the most abundant ASV in the entire dataset, accounting for 5.8% of the total sequences in wild samples. This ASV was classified only to the order *Bacteroidales*, with only 86.7% nucleotide identity to the nearest reference. Overall, these results suggest that captivity has a major influence on SHNW gut microbiota diversity and composition, and that the SHNW gut contains bacteria that are highly divergent from current database references.

### Population-level gut microbiota signatures in the SHNW

Next, we focused on comparing and contrasting microbial composition between different wild SHNW populations, which include differences in habitat (Wonga has intact native grasses, forbs and chenopods, whereas Kooloola and Brookfield represent degraded pastoral habitat with introduced weed species comprising most of the SHNWs diet).

**Fig. 4:**
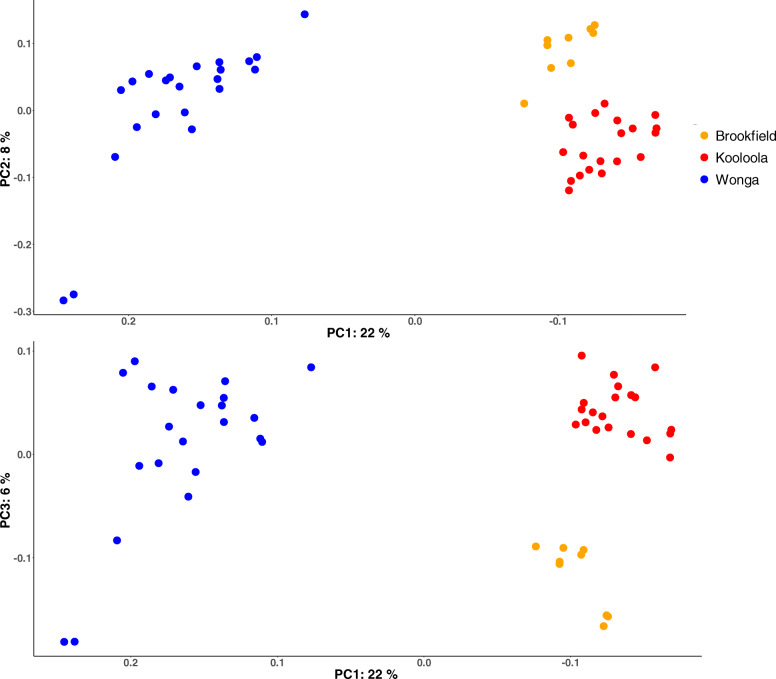
Differences in microbial composition between the three wild SHNW populations Wonga (intact habitat), Kooloola and Brookfield (degraded habitat). PERMANOVA of microbial composition between populations is statistically significant. Principal coordinates analysis (PCOA) of unweighted UniFrac distances show separation of Wonga and Kooloola/Brookfield samples on PC1. PC2 and PC3 appear to separate Kooloola and Brookfield samples

Samples from Wonga were clearly separated from Brookfield and Kooloola along the first principal coordinate, which explained 22% of the variation (Fig. 4), and these differences in microbial composition were statistically significant (PERMANOVA of unweighted UniFrac distances *p*-values = 0.001, pseudo-F = 12.1 and 7.9 for Wonga vs. Kooloola and Wonga vs. Brookfield, respectively). Samples from Kooloola and Brookfield were also separated from each other across principal coordinates 2 and 3, which explained 8 and 6% of the variation, respectively, and these differences were also statistically significant (PERMANOVA of unweighted UniFrac distance *p*-value = 0.001, pseudo-F = 4.3). These results were also similar when using the weighted Unifrac metric, with the exception of the differences between Brookfield and Kooloola samples (SI Fig. 4).

**Fig. 5:**
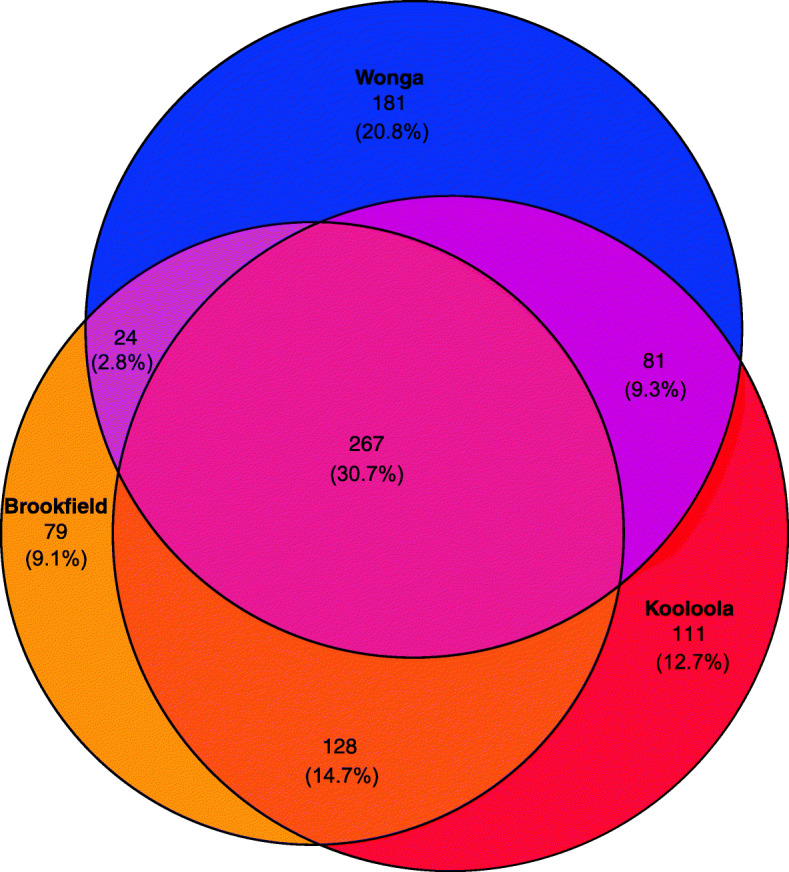
Venn diagram of ASVs shared and unique between different SHNW populations, with the percentage of total ASVs listed for each region. Wonga (intact habitat), Kooloola and Brookfield (degraded habitats)

Using ANCOM, we found 7 families that had differential abundance between wild populations (SI File 4). *Streptococcaceae* had a higher relative abundance in Kooloola samples, an unclassified *RF39* family was only found in Kooloola and Brookfield samples, and *Micrococcaceae* had a higher relative abundance in Wonga samples. At the sequence level, 14 ASVs were significantly differentially abundant between populations (SI File 5). Between the different SHNW populations, 111 ASVs were found only in Kooloola samples (accounting for 7.6% total abundance), 79 were found only in Brookfield samples (4%), 181 ASVs were found only in the Wonga samples (10.9%), and 267 were shared in all wild samples (Fig. 5). To test whether these population-specific ASVs were due to differences in soil microbiota between sampling sites, we ran SourceTracker2 with the soil samples set as sources and the faecal samples set as sinks. We found that soil microbes were not a major component of the SHNW faecal samples (mixing proportions < 0.001; SI Table 4).

## Discussion

Captive management of wild mammals is typically accompanied by a drastic shift in environment and diet, factors that have been shown to have a large influence on the gut microbiota of mammals. In addition, different populations of the same animal species have been found to harbour unique gut microbial communities, especially across distinct habitats [14, 15]. These findings spurred us to investigate their relevance to the gut microbiota of the SHNW, a species of conservation significance [24]. We found that captive SHNWs had reduced gut microbiota diversity and a substantially different microbial composition compared to wild SHNWs. We also found population-specific differences in microbial composition between the three wild SHNW populations surveyed, with the largest differences occurring between SHNWs living in intact versus degraded habitats.

The gut microbiota of mammalian herbivores provides key functions involved in the extraction of nutrients and energy from the diet that would otherwise be unavailable to the host [52, 53]. This reliance of herbivorous mammals on their gut microbial communities has resulted in physiological and morphological adaptations of the mammalian gastrointestinal tract to enhance the extraction of energy by the gut microbiota, including an increase in length and capacity [54]. The SHNW is a hindgut fermenter with an extremely long and capacious gastrointestinal tract (~ 12 times body length) [21]. As the diet of SHNWs largely consists of plant cell walls that are recalcitrant to host enzymes, SCFAs produced through the microbial degradation and fermentation of these compounds in the colon are thought to form the bulk (> 60%) of the daily energy requirements of SHNWs [6]. Previous metagenomic functional profiling of a single captive SHNW found microbial capacity for the degradation of complex plant polysaccharides, including cellulose, xylan, and hemicellulose [55]. While such functions were found in a captive SHNW, we would predict that captivity reduces the diversity of such genes, which could impact the ability of SHNWs to derive energy from wild diets. Disruptions to the gut microbiota of SHNWs could therefore have major negative impacts to their health and fitness.

Our finding that captive SHNWs harboured reduced microbial diversity and substantially different microbial composition compared to wild SHNWs follows the general trend previously observed in other mammalian species [7, 9]. At the sequence level we observed major differences, with only 7.8% of ASVs being shared between captive and wild SHNWs -- accounting for only 26.3 and 28.7% of their gut microbiota, respectively. The marked difference in diet could be a major contributing factor, with captive SHNWs in our study being fed a more digestible diet. Such a diet could select for a gut microbiota adapted to simple sugars and starch, with less reliance on complex metabolic pathways involved in the degradation and fermentation of resistant polysaccharides. This has been previously observed in humans, with Italian children consuming a diet containing less fibre and more starches having lower diversity and depleted levels of microbes associated with complex carbohydrate metabolism compared to children living in Burkina Faso who consumed a diet high in fibre [56]. This has also been demonstrated experimentally in laboratory mice, with a loss of microbial diversity being linked to a reduction of dietary fibre [12].

Like many other mammals [57], the SHNW gut microbiota was dominated (> 70%) by the phyla *Firmicutes* and *Bacteroidetes*. We observed substantial differences in the relative abundance of these phyla between captive and wild SHNWs, with captive animals having a mean *Firmicutes*:*Bacteroidetes* ratio of 4.8:1 vs. 1.7–0.8:1 for wild populations. This could be due to the differences in availability and types of fibre present in captive and wild diets, as there are species within the *Bacteroidetes* and *Firmicutes* phyla that are known to harbour diverse carbohydrate active enzymes [58]. In humans, an increase in *Bacteroidetes* and decrease in *Firmicutes* was observed in children consuming larger quantities and diversity of fibre [56]. Mice fed a diet low in fibre also preferentially lost microbes belonging to the phylum *Bacteroidetes* [12]. Such changes in abundance or loss may result in a reduced capacity to produce SCFAs. Children eating less fibre had significantly lower concentrations of SCFA measured in faeces compared to children on a fibre-rich diet [56]. Barboza & Hume also found that wild SHNWs produced substantially more SCFAs than captive animals, with wild animals also producing greater quantities of propionate and reduced quantities of butyrate compared to captive held SHNWs [6].

However, caution should be used when interpreting the putative functions of microbes based on their taxonomy, particularly in our dataset, as the microbes of Australian marsupials remain little studied. Many of the microbes classified in this study were distantly related to characterised microbes—the most abundant ASV had only 86.7% nucleotide identity to the closest reference database sequence. Further genome-centric and biochemical analyses will be required to test the hypothesis that captivity results in a loss of microbial metabolic potential to degrade and ferment complex plant polysaccharides in SHNWs. Overall, our findings suggest that captivity results in a loss of microbial diversity and marked shifts in the gut microbiota composition of SHNWs. Such changes could reduce the energy economy of SHNWs being translocated from captivity to the wild. While the SHNW is only considered “Near-Threatened” at present, its close relative the Northern Hairy-nosed Wombat (*Lasiorhinus krefftii*) is listed as critically endangered, with an estimated population size < 300 [59]. Future captive breeding programs for this species should take precautionary steps to reduce the loss of wild gut microbiota diversity in captivity, such as the feeding of natural diets [13].

We also observed significant differences in microbial composition between the three different wild SHNW populations sampled, with the largest difference being between Wonga (intact habitat) versus Brookfield and Kooloola (degraded habitats). One possible reason for this finding is that differences in floral assemblages between intact/degraded habitats selected for distinct gut microbial compositions that were better adapted to deriving energy from the different plant species. Previous work on herbivorous primates identified changes in gut microbial diversity and composition between populations from habitats of differing quality [14, 15], with animals from native habitats possessing a greater number of genes associated with SCFA production and hydrogen metabolism [14]. These differences also align with significant differences in body condition score between wombats from Wonga and Kooloola across all cohorts, with animals from Wonga (intact habitat) in significantly better condition, at all times of the year, than those from Kooloola (degraded habitat) (Taggart et al. in preparation). The implications of these findings on breeding, survival of young, and resilience to drought in their semi-arid environment are likely to be significant. Another possible reason for these differences could relate to the presence of toxic plant species in the degraded habitats (Brookfield/Kooloola). These degraded habitats harboured plant species including Potato weed, Ward’s weed, and Onion weed, and contain toxic plant defence compounds (eg. pyrrolizidine alkaloids) [31, 60]. Previous work on other herbivorous mammals has demonstrated that the gut microbiota can detoxify plant defence compounds for the host [61–63]. Therefore, some of the gut microbes identified as unique to SHNWs living in degraded habitats could play roles in the detoxification of plant defence compounds. To test these possible factors, shotgun metagenomics could be used to identify genes or pathways associated with the degradation of different plant cell wall components, or the detoxification of plant defence compounds.

Seasonal variation (e.g. temperature, rainfall and food availability) can play a major role in shaping the gut microbiota of animals [64]. As seasons affect plant assemblages in these habitats (e.g. grasses tend to emerge after adequate rainfall, and subside in drier months), sampling the same wombat populations at different times of the year could yield insights into how the gut microbiota is changing or adapting to host diet. The South Australian Murraylands, where this study was undertaken, has a semi-arid environment with most rainfall and associated plant growth occurring in winter and spring followed by long, hot and dry summer and autumn months. In this study, wombat faecal samples from degraded habitats (Kooloola and Brookfield) were collected in March (autumn) and those from the intact habitat (Wonga) were collected 3 months later in mid June (start of winter). However, in 2019 when this study was conducted the region was in drought with little rainfall falling in the first half of the year, and minimal rainfall (5–7 mm / month) recorded prior to sampling at any of the sites we investigated: Kooloola 14 mm between Jan - March, (BOM, Swan Reach); Brookfield 20 mm between Jan-March (BOM, Blanchtown); and Wonga 45.6 mm between Jan - mid June (BOM, Robertstown). As a consequence, rainfall associated vegetation changes were also minimal at these sites. Differences in sampling times in this study are thus unlikely to drive the differences in wombat gut microbiomes observed between degraded and intact landscapes. Finally, host sex has been shown to influence the gut microbiota of vertebrates [65], and while our study could not determine the sex of wild animals, it appears that capitivity and population are more important drivers of the SHNW gut microbiota.

An alternate explanation for the differences in SHNW gut microbiota composition between wild populations is neutral allopatric speciation of microbes with their hosts [66]. As populations separate from each other the opportunity for microbes to be shared between them is reduced. Microbes within each separate population can then continue to acquire mutations and co-speciate with their host, leading to observed differences between populations. Dispersal in SHNWs is thought to be limited and biased toward females [27, 67], with most animals staying within several hundred metres of their burrow of birth. Brookfield and Kooloola are physically closer to each other than to Wonga station. Therefore, the observed differences in gut microbiota composition between wild SHNW populations could be a product of minimal dispersal of animals between the populations, precluding the sharing of microbes between populations. Future host genetic investigations into the population structure and history of SHNWs in South Australia would provide a scaffold for which to test whether the gut microbiota is allopatrically speciating. Additionally, greater sampling of SHNW gut microbiota across their entire distribution (including the Nullarbor, which is ~ 1000 km west of the populations tested here) will also be needed to test this scenario.

## Conclusions

We found that captivity significantly altered the diversity and composition of the gut microbiota in SHNWs. We also detected population-specific microbial compositions in the gut microbiota of wild SHNWs, which could represent microbial adaptations to the different habitats. How important these microbial differences are to host health and fitness remains to be determined, but could have management implications for SHNWs in the future, such as modifying captive diets or using faecal microbiota transplants to assist with SHNW translocations.

## Supplementary Information

**Additional file 1:** **SI_Figure_1**. PCoA plot of unweighted UniFrac distances including the 7 outlier Brookfield samples.

**Additional file 2:** **SI_Figure_2**. Phylum-level taxonomic barplots including the 7 outlier Brookfield samples.

**Additional file 3:** **SI_Figure_3**. PCoA plots of weighted UniFrac distances for captive and wild samples.

**Additional file 4:** **SI_Figure_4**. PCoA plots of weighted UniFrac distances for the different populations of wild samples.

**Additional file 5:** **SI_Table_1**. Pairwise Kruskal-Wallis test results for Faith’s phylogenetic diversity metric comparisons between populations.

**Additional file 6:** **SI_Table_2**. Pairwise Kruskal-Wallis test results for observed features (richness) metric comparisons between populations.

**Additional file 7:** **SI_Table_3**. SINA alignment results of the most abundant core ASVs in SHNWs.

**Additional file 8:** **SI_Table_4**. SourceTracker2 results testing the estimated proportion of faecal bacteria coming from soil. https://doi.org/10.25909/12979157.

**Additional file 9:** **SI_File_1**. QIIME2 qzv file of taxonomic bar plots per sample.

**Additional file 10:** **SI_File_2**. QIIME2 qzv file of ANCOM test at family level (captive and wild samples).

**Additional file 11:** **SI_File_3**. QIIME2 qzv file of ANCOM test at the ASV level (captive and wild samples).

**Additional file 12:** **SI_File_4**. QIIME2 qzv file of ANCOM test at family level (between different wild populations).

**Additional file 13:** **SI_File_5**. QIIME2 qzv file of ANCOM test at the ASV level (between different wild populations).

## Data Availability

Raw sequencing data are available at the NCBI SRA (BioProject ID PRJNA663974). All QIIME2, code, analysis files, and R code used to plot figures can be found here: https://github.com/EisenRa/2020_SHNW_Faecal_16S. All files have also been frozen at the following DOI: https://zenodo.org/badge/latestdoi/316123393.

## Abbreviations

ASV: Amplicon Sequence Variant
HTS: High-Throughput Sequencing
PCoA: Principal Coordinates Analysis
PCR: Polymerase Chain Reaction
SCFA: Short-Chain Fatty Acid
SHNW: Southern Hairy-nosed Wombat
