# Supplementary figures and images for: Signatures of landscape and captivity in the gut microbiota of Southern Hairy-nosed Wombats (Lasiorhinus latifrons)
Source: Anim Microbiome. 2021 Jan 6;3:4. doi: 10.1186/s42523-020-00068-y (PMC7934541; doi:10.1186/s42523-020-00068-y)

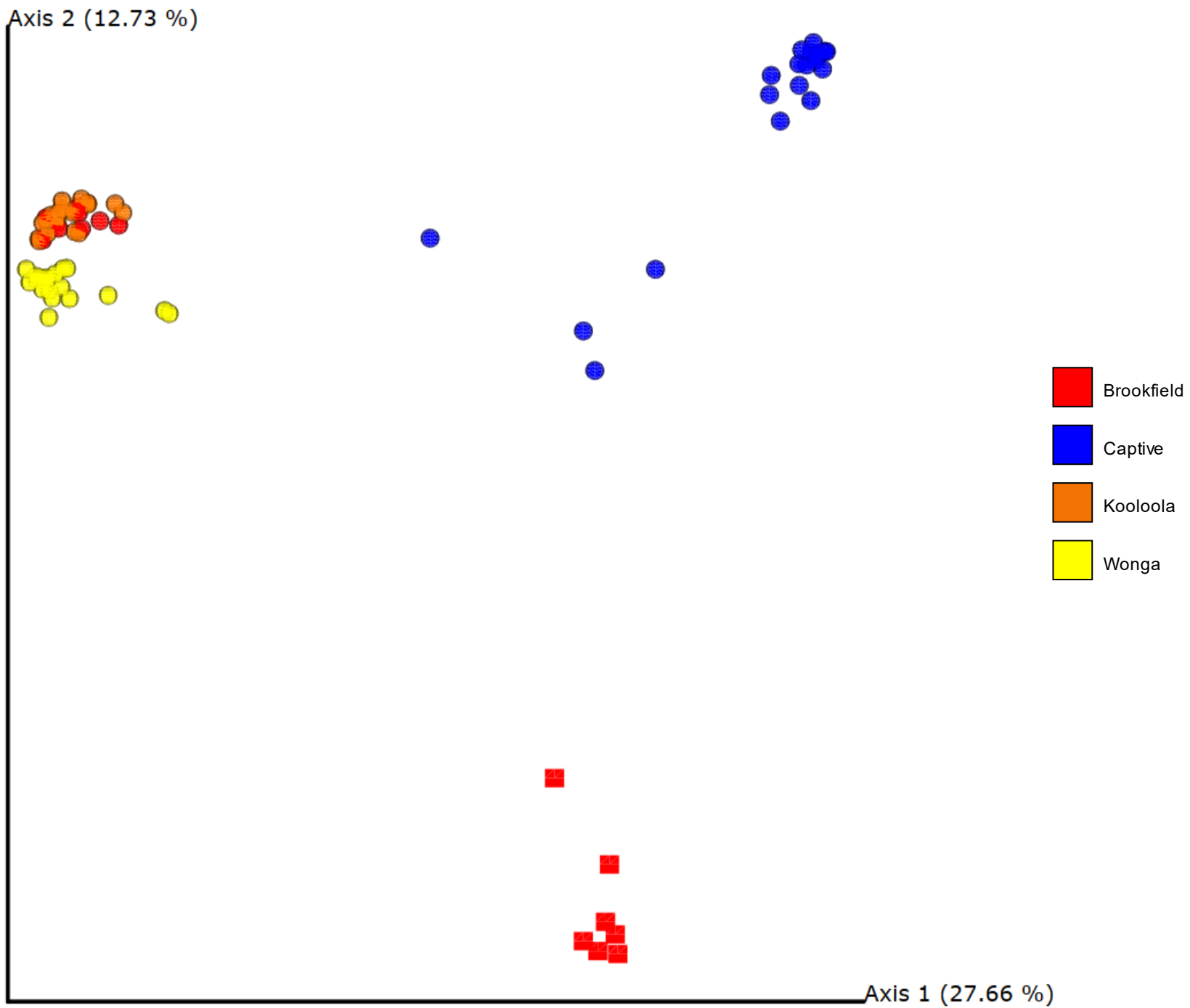

Supplement: Supplementary file 1 — Additional file 1: SI_Figure_1. PCoA plot of unweighted UniFrac distances including the 7 outlier Brookfield samples. [file 42523_2020_68_MOESM1_ESM.pdf]

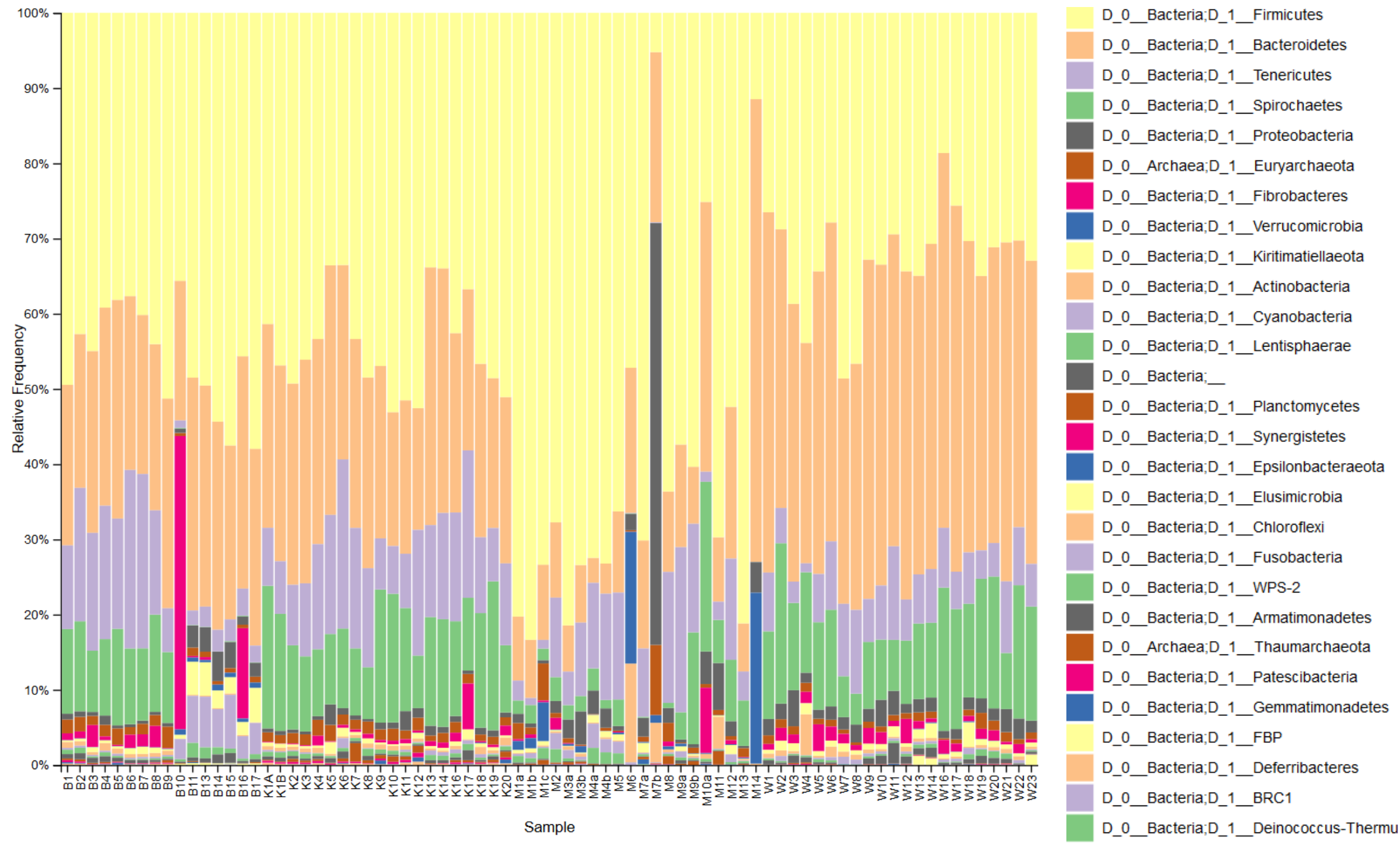

Supplement: Supplementary file 2 — Additional file 2: SI_Figure_2. Phylum-level taxonomic barplots including the 7 outlier Brookfield samples. [file 42523_2020_68_MOESM2_ESM.pdf]

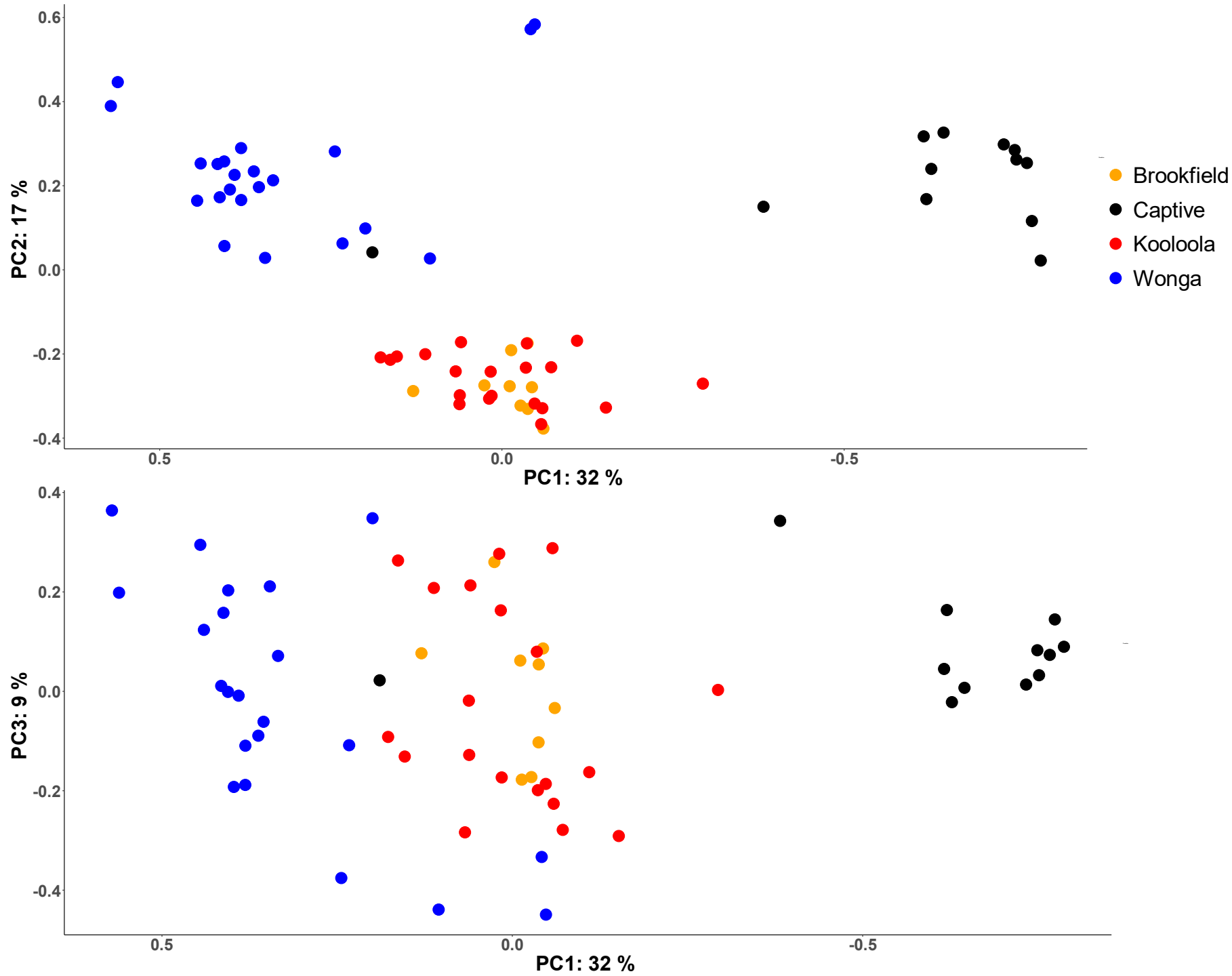

Supplement: Supplementary file 3 — Additional file 3: SI_Figure_3. PCoA plots of weighted UniFrac distances for captive and wild samples. [file 42523_2020_68_MOESM3_ESM.pdf]

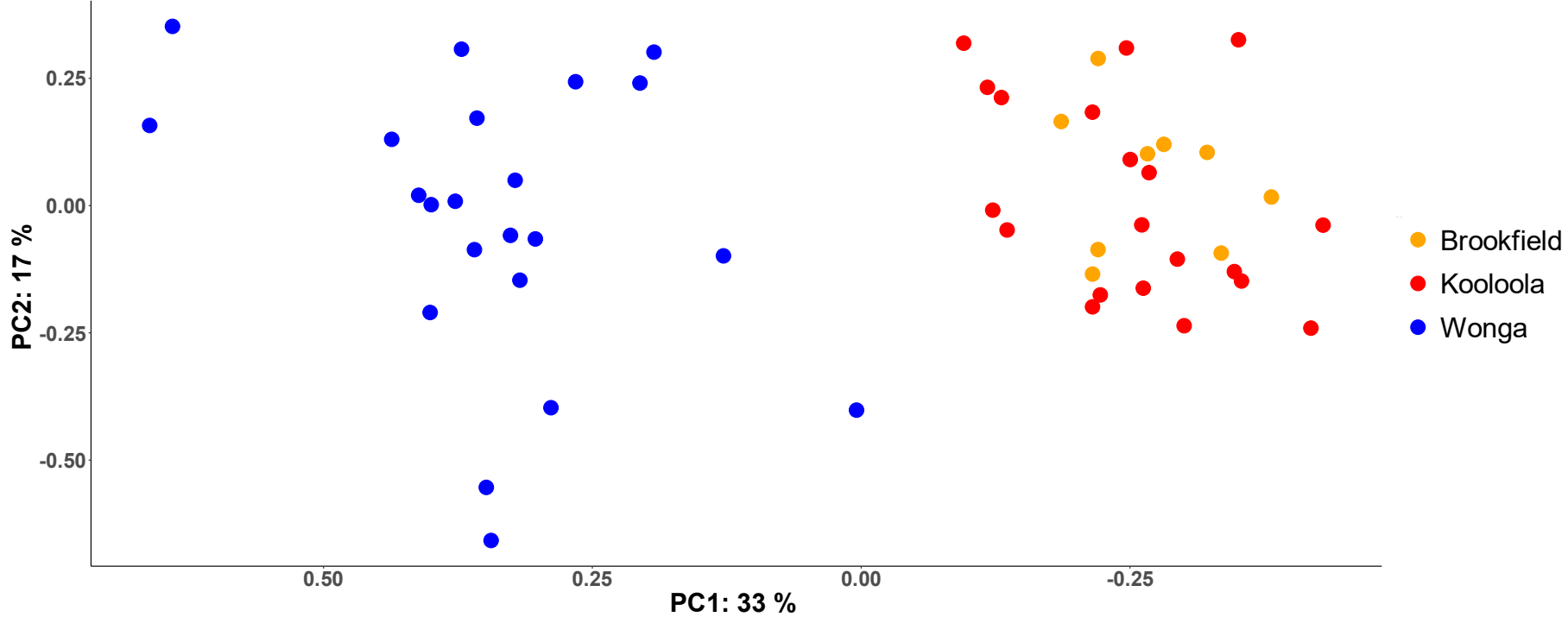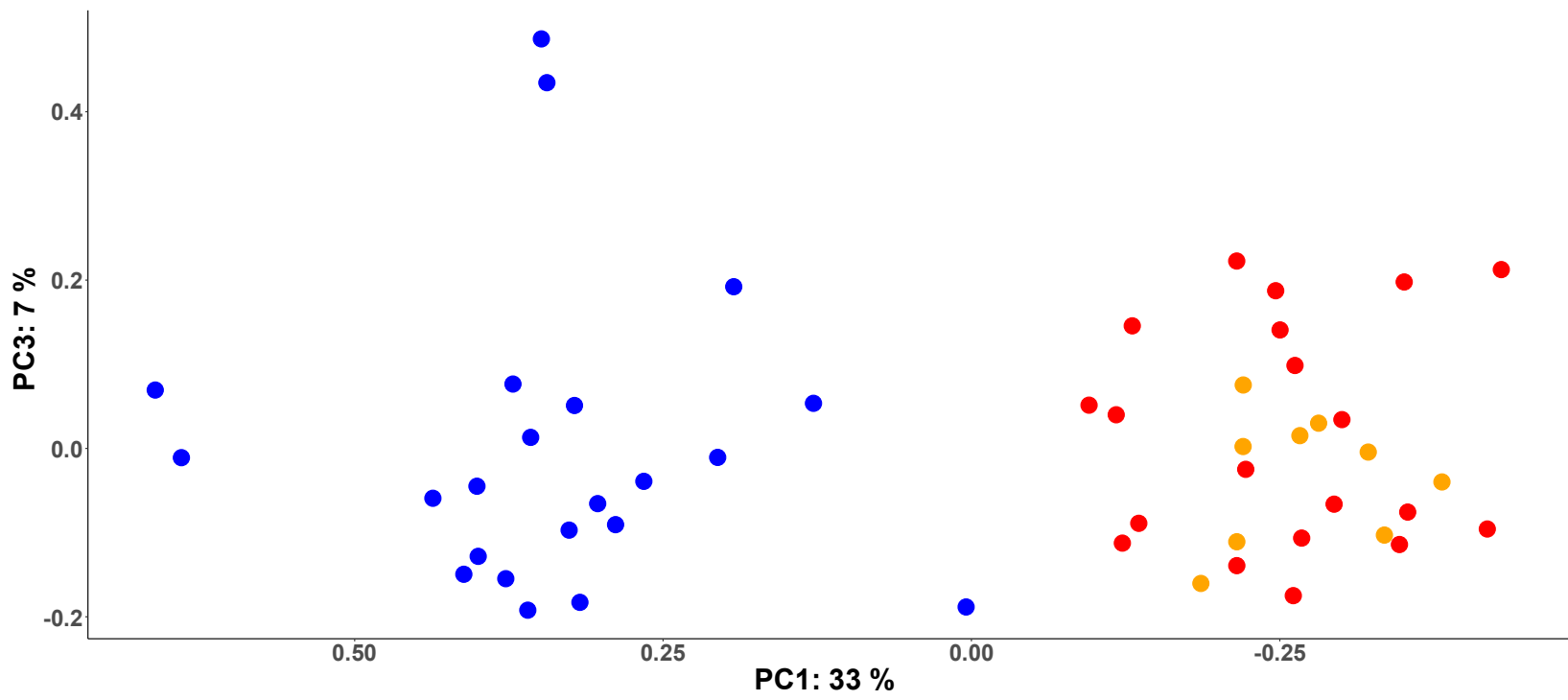

Supplement: Supplementary file 4 — Additional file 4: SI_Figure_4. PCoA plots of weighted UniFrac distances for the different populations of wild samples. [file 42523_2020_68_MOESM4_ESM.pdf]

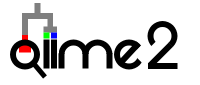

Supplement: Supplementary file 9 — Additional file 9: SI_File_1. QIIME2 qzv file of taxonomic bar plots per sample. [file 42523_2020_68_MOESM9_ESM.qzv › 854a8f14-b509-4353-8f4a-4b6549c285ec/data/q2templateassets/img/qiime2-rect-200.png]
