# Supplementary material for: Signatures of landscape and captivity in the gut microbiota of Southern Hairy-nosed Wombats (Lasiorhinus latifrons)
Source: Anim Microbiome. 2021 Jan 6;3:4. doi: 10.1186/s42523-020-00068-y (PMC7934541; doi:10.1186/s42523-020-00068-y)
Supplement: Supplementary file 10 — Additional file 10: SI_File_2. QIIME2 qzv file of ANCOM test at family level (captive and wild samples). [file 42523_2020_68_MOESM10_ESM.qzv › f6ed9c16-6785-46af-9f96-24e084e80892/data/index.html]

q2\_composition : ancom


### ANCOM Volcano Plot

---

#### ANCOM statistical results

|  | W |
| --- | --- |
| D\_0\_\_Bacteria;D\_1\_\_Lentisphaerae;D\_2\_\_Lentisphaeria;D\_3\_\_Victivallales;D\_4\_\_vadinBE97 | 109 |
| D\_0\_\_Bacteria;D\_1\_\_Bacteroidetes;D\_2\_\_Bacteroidia;D\_3\_\_Bacteroidales;D\_4\_\_p-2534-18B5 gut group | 105 |
| D\_0\_\_Bacteria;D\_1\_\_Cyanobacteria;D\_2\_\_Melainabacteria;D\_3\_\_Gastranaerophilales;D\_4\_\_uncultured bacterium | 103 |
| D\_0\_\_Bacteria;D\_1\_\_Kiritimatiellaeota;D\_2\_\_Kiritimatiellae;D\_3\_\_WCHB1-41;D\_4\_\_metagenome | 100 |
| D\_0\_\_Bacteria;D\_1\_\_Kiritimatiellaeota;D\_2\_\_Kiritimatiellae;D\_3\_\_WCHB1-41;D\_4\_\_uncultured bacterium | 100 |
| D\_0\_\_Bacteria;D\_1\_\_Bacteroidetes;D\_2\_\_Bacteroidia;D\_3\_\_Bacteroidales;D\_4\_\_Marinifilaceae | 98 |
| D\_0\_\_Bacteria;D\_1\_\_Firmicutes;D\_2\_\_Bacilli;D\_3\_\_Lactobacillales;D\_4\_\_Lactobacillaceae | 98 |
| D\_0\_\_Bacteria;D\_1\_\_Bacteroidetes;D\_2\_\_Bacteroidia;D\_3\_\_Bacteroidales;D\_4\_\_p-251-o5 | 97 |
| D\_0\_\_Bacteria;D\_1\_\_Bacteroidetes;D\_2\_\_Bacteroidia;D\_3\_\_Bacteroidales;D\_4\_\_F082 | 97 |
| D\_0\_\_Archaea;D\_1\_\_Euryarchaeota;D\_2\_\_Methanomicrobia;D\_3\_\_Methanomicrobiales;D\_4\_\_Methanocorpusculaceae | 96 |
| D\_0\_\_Bacteria;D\_1\_\_Bacteroidetes;D\_2\_\_Bacteroidia;\_\_;\_\_ | 95 |
| D\_0\_\_Bacteria;D\_1\_\_Firmicutes;D\_2\_\_Bacilli;D\_3\_\_Lactobacillales;D\_4\_\_Leuconostocaceae | 94 |
| D\_0\_\_Bacteria;D\_1\_\_Lentisphaerae;D\_2\_\_Oligosphaeria;D\_3\_\_Oligosphaerales;D\_4\_\_Oligosphaeraceae | 93 |
| D\_0\_\_Bacteria;D\_1\_\_Firmicutes;D\_2\_\_Bacilli;D\_3\_\_Lactobacillales;D\_4\_\_Enterococcaceae | 92 |
| D\_0\_\_Bacteria;D\_1\_\_Proteobacteria;\_\_;\_\_;\_\_ | 91 |
| D\_0\_\_Bacteria;D\_1\_\_Tenericutes;D\_2\_\_Mollicutes;D\_3\_\_Izimaplasmatales;D\_4\_\_Izimaplasmataceae | 90 |
| D\_0\_\_Bacteria;D\_1\_\_Bacteroidetes;D\_2\_\_Bacteroidia;D\_3\_\_Sphingobacteriales;D\_4\_\_Sphingobacteriaceae | 89 |
| D\_0\_\_Bacteria;D\_1\_\_Bacteroidetes;D\_2\_\_Bacteroidia;D\_3\_\_Bacteroidales;\_\_ | 88 |
| D\_0\_\_Bacteria;D\_1\_\_Cyanobacteria;D\_2\_\_Melainabacteria;D\_3\_\_Gastranaerophilales;D\_4\_\_uncultured rumen bacterium | 87 |
| D\_0\_\_Bacteria;D\_1\_\_Bacteroidetes;D\_2\_\_Bacteroidia;D\_3\_\_Bacteroidales;D\_4\_\_Marinilabiliaceae | 85 |
| D\_0\_\_Bacteria;D\_1\_\_Verrucomicrobia;D\_2\_\_Verrucomicrobiae;D\_3\_\_Opitutales;D\_4\_\_Puniceicoccaceae | 83 |

Download table as TSV

---

#### Percentile abundances of features by group

| Percentile | 0.0 | 25.0 | 50.0 | 75.0 | 100.0 | 0.0 | 25.0 | 50.0 | 75.0 | 100.0 |
| --- | --- | --- | --- | --- | --- | --- | --- | --- | --- | --- |
| Group | No | No | No | No | No | Yes | Yes | Yes | Yes | Yes |
| D\_0\_\_Bacteria;D\_1\_\_Lentisphaerae;D\_2\_\_Lentisphaeria;D\_3\_\_Victivallales;D\_4\_\_vadinBE97 | 1.0 | 1.0 | 1.0 | 1.0 | 5.0 | 31.0 | 178.75 | 346.5 | 938.25 | 1180.0 |
| D\_0\_\_Bacteria;D\_1\_\_Bacteroidetes;D\_2\_\_Bacteroidia;D\_3\_\_Bacteroidales;D\_4\_\_p-2534-18B5 gut group | 1.0 | 1.0 | 1.0 | 1.0 | 4.0 | 1.0 | 148.75 | 330.0 | 587.25 | 1625.0 |
| D\_0\_\_Bacteria;D\_1\_\_Cyanobacteria;D\_2\_\_Melainabacteria;D\_3\_\_Gastranaerophilales;D\_4\_\_uncultured bacterium | 1.0 | 1.0 | 1.0 | 1.0 | 48.0 | 1.0 | 77.50 | 217.0 | 773.00 | 1942.0 |
| D\_0\_\_Bacteria;D\_1\_\_Kiritimatiellaeota;D\_2\_\_Kiritimatiellae;D\_3\_\_WCHB1-41;D\_4\_\_metagenome | 1.0 | 1.0 | 1.0 | 1.0 | 1.0 | 1.0 | 31.00 | 271.0 | 763.25 | 1929.0 |
| D\_0\_\_Bacteria;D\_1\_\_Kiritimatiellaeota;D\_2\_\_Kiritimatiellae;D\_3\_\_WCHB1-41;D\_4\_\_uncultured bacterium | 73.0 | 253.0 | 394.0 | 700.5 | 2725.0 | 1.0 | 1.00 | 1.0 | 1.00 | 33.0 |
| D\_0\_\_Bacteria;D\_1\_\_Bacteroidetes;D\_2\_\_Bacteroidia;D\_3\_\_Bacteroidales;D\_4\_\_Marinifilaceae | 1.0 | 1.0 | 1.0 | 1.0 | 53.0 | 1.0 | 35.25 | 128.0 | 181.00 | 898.0 |
| D\_0\_\_Bacteria;D\_1\_\_Firmicutes;D\_2\_\_Bacilli;D\_3\_\_Lactobacillales;D\_4\_\_Lactobacillaceae | 1.0 | 1.0 | 1.0 | 3.0 | 9.0 | 1.0 | 50.00 | 120.0 | 217.75 | 371.0 |
| D\_0\_\_Bacteria;D\_1\_\_Bacteroidetes;D\_2\_\_Bacteroidia;D\_3\_\_Bacteroidales;D\_4\_\_p-251-o5 | 1.0 | 1823.0 | 3715.0 | 8205.0 | 29093.0 | 3.0 | 7.50 | 12.0 | 17.25 | 20.0 |
| D\_0\_\_Bacteria;D\_1\_\_Bacteroidetes;D\_2\_\_Bacteroidia;D\_3\_\_Bacteroidales;D\_4\_\_F082 | 1.0 | 462.5 | 1090.0 | 1699.5 | 3572.0 | 1.0 | 1.00 | 1.0 | 3.00 | 3095.0 |
| D\_0\_\_Archaea;D\_1\_\_Euryarchaeota;D\_2\_\_Methanomicrobia;D\_3\_\_Methanomicrobiales;D\_4\_\_Methanocorpusculaceae | 3.0 | 438.5 | 605.0 | 988.5 | 4431.0 | 1.0 | 1.00 | 1.0 | 3.00 | 141.0 |
| D\_0\_\_Bacteria;D\_1\_\_Bacteroidetes;D\_2\_\_Bacteroidia;\_\_;\_\_ | 326.0 | 1034.0 | 2159.0 | 5464.5 | 21066.0 | 1.0 | 6.50 | 12.0 | 15.00 | 561.0 |
| D\_0\_\_Bacteria;D\_1\_\_Firmicutes;D\_2\_\_Bacilli;D\_3\_\_Lactobacillales;D\_4\_\_Leuconostocaceae | 1.0 | 1.0 | 1.0 | 1.0 | 1.0 | 1.0 | 9.00 | 15.5 | 55.75 | 154.0 |
| D\_0\_\_Bacteria;D\_1\_\_Lentisphaerae;D\_2\_\_Oligosphaeria;D\_3\_\_Oligosphaerales;D\_4\_\_Oligosphaeraceae | 13.0 | 153.0 | 254.0 | 377.5 | 2165.0 | 1.0 | 1.00 | 1.0 | 1.00 | 263.0 |
| D\_0\_\_Bacteria;D\_1\_\_Firmicutes;D\_2\_\_Bacilli;D\_3\_\_Lactobacillales;D\_4\_\_Enterococcaceae | 3.0 | 5.0 | 7.0 | 13.0 | 105.0 | 5.0 | 35.75 | 100.0 | 417.50 | 1292.0 |
| D\_0\_\_Bacteria;D\_1\_\_Proteobacteria;\_\_;\_\_;\_\_ | 1.0 | 1.0 | 1.0 | 1.0 | 3.0 | 1.0 | 3.25 | 28.0 | 64.50 | 1309.0 |
| D\_0\_\_Bacteria;D\_1\_\_Tenericutes;D\_2\_\_Mollicutes;D\_3\_\_Izimaplasmatales;D\_4\_\_Izimaplasmataceae | 1.0 | 714.5 | 2270.0 | 5557.5 | 23843.0 | 3.0 | 3.75 | 5.5 | 8.75 | 13.0 |
| D\_0\_\_Bacteria;D\_1\_\_Bacteroidetes;D\_2\_\_Bacteroidia;D\_3\_\_Sphingobacteriales;D\_4\_\_Sphingobacteriaceae | 1.0 | 426.5 | 913.0 | 2025.5 | 9333.0 | 1.0 | 1.00 | 3.5 | 5.25 | 2780.0 |
| D\_0\_\_Bacteria;D\_1\_\_Bacteroidetes;D\_2\_\_Bacteroidia;D\_3\_\_Bacteroidales;\_\_ | 140.0 | 399.5 | 704.0 | 1091.0 | 6473.0 | 1.0 | 1.00 | 2.0 | 57.75 | 178.0 |
| D\_0\_\_Bacteria;D\_1\_\_Cyanobacteria;D\_2\_\_Melainabacteria;D\_3\_\_Gastranaerophilales;D\_4\_\_uncultured rumen bacterium | 1.0 | 1.0 | 1.0 | 1.0 | 3.0 | 1.0 | 1.00 | 7.0 | 84.50 | 533.0 |
| D\_0\_\_Bacteria;D\_1\_\_Bacteroidetes;D\_2\_\_Bacteroidia;D\_3\_\_Bacteroidales;D\_4\_\_Marinilabiliaceae | 1.0 | 34.5 | 95.0 | 502.5 | 4899.0 | 1.0 | 1.00 | 1.0 | 1.00 | 3.0 |
| D\_0\_\_Bacteria;D\_1\_\_Verrucomicrobia;D\_2\_\_Verrucomicrobiae;D\_3\_\_Opitutales;D\_4\_\_Puniceicoccaceae | 1.0 | 1.0 | 1.0 | 23.0 | 145.0 | 12.0 | 31.50 | 114.0 | 153.50 | 805.0 |

Download table as TSV
