# Supplementary material for: Signatures of landscape and captivity in the gut microbiota of Southern Hairy-nosed Wombats (Lasiorhinus latifrons)
Source: Anim Microbiome. 2021 Jan 6;3:4. doi: 10.1186/s42523-020-00068-y (PMC7934541; doi:10.1186/s42523-020-00068-y)
Supplement: Supplementary file 11 — Additional file 11: SI_File_3. QIIME2 qzv file of ANCOM test at the ASV level (captive and wild samples). [file 42523_2020_68_MOESM11_ESM.qzv › be699df9-32b3-4a7b-b107-85e93c89e28f/data/index.html]

q2\_composition : ancom


### ANCOM Volcano Plot

---

#### ANCOM statistical results

|  | W |
| --- | --- |
| bda6b6e7da7508c68057140e7bd6cd8d | 982 |
| c64278b7c0c20b3412e0cf6b3bd546fb | 955 |
| 9171cba1631b9a713a9d4c2e1c3408f0 | 946 |
| ee2e7e1ec3775388a6a1000c5de4c483 | 928 |
| 7ab1bc6052053650189083370055ae66 | 923 |
| 3b092a59cd0e2d57483420cc994ec49c | 922 |
| b1a153ff5a21342991138e7f7dd2658e | 915 |
| 0fb0f66b7e335cc4480b4f827e958ab3 | 910 |
| d061a99148d649c4eca4836c5f4b5b68 | 909 |
| 0ac7d4cacbb81c9c8182426c988a1c21 | 907 |
| 7b0c4c30e3e9348105221123952bf547 | 899 |
| 8c8c1f03f8c009f91e286e431aa80623 | 894 |
| 07d05a9cba0df11f278b17a0ea24eed3 | 893 |
| 2ca99e2c6c0fcb152b4919fa00c4eeed | 880 |
| 9ee8e39c88b826bd1bfc99589655f49d | 877 |
| 3b17bc4edbddbe11a9a573e5eebf8d66 | 877 |
| 9dd9cefe38347b087dd318d82962cc03 | 875 |
| b696a6a2ff1f6de34e946662a88c6a24 | 875 |
| e18e57dcbb611101406f8fb1d180b8a1 | 871 |
| b902443fbf36d9390d212daef4f9bb11 | 871 |
| b89075e3356afd5634655288c5a3d1b8 | 870 |
| 7de0b23e91fd1db3bfb5b37cb92936ae | 860 |
| 1a494aa43d60de2b0130682daa0c8dd1 | 857 |
| 2c5c5d710e1b9229efa72f98ca7b4e71 | 854 |
| 8ebe701cf6fe9fecac2a0d2ba0f74884 | 851 |
| 14988fea81d67867717b050e6b892e63 | 849 |
| b423592b0ae631882095792b27729b9c | 843 |
| cd688ebb10111cd2be59c7eb5fed07a3 | 842 |
| e6924b794a53c647f1a9509519f37c9a | 840 |
| 599ba6458e91e2527f358f547ea39261 | 839 |
| ac06b2ac668f171362228caae9c12747 | 839 |
| 08dd9f8036267148e2dc831fbb594689 | 839 |
| beef5a54aaee13f217ecede427cb3071 | 838 |
| 9c9210d344e78f933f4aa6da483f6683 | 836 |
| 6436532cd975b8c8000f486deac9fb51 | 828 |
| e072125a988d90ca4d1c96a5ef091210 | 826 |
| e91f1c5b67ffecb5ca3ca3b393a50f00 | 821 |
| 964f4677a8ea31cce60d46326065323b | 820 |
| ecaa9f4e15a28f212d9bd60f749191ab | 820 |
| c79b3d87e705bf6859d8b3a8a57577f9 | 818 |
| 34584166f11d66d5f290bc19bc44edc4 | 818 |
| 957902900b34c7f1ec534fbd5c29b77a | 818 |
| 3996fdc07a002f768922b79975cae51c | 815 |
| 6c36809e5d98ab855c5ef2b8534596d3 | 813 |
| 8743c146ab4737302c555e0b4f96f710 | 812 |
| 56758836e2fc766051d575e08978cdec | 812 |
| b0645cbec7653c887cb868a248d95874 | 808 |
| b70073c1864e1f977594ed75f642df15 | 807 |
| 0021d135d4ac12982cc8abdf2b38e23f | 805 |
| 476ee1e58388dfab6f1796baab4dc203 | 803 |
| e11cb2911aeaeb30eb0041b063506871 | 801 |
| 7f8b90a32aa7cec1f34061cad0adc662 | 801 |
| cae14798faeabb8e1b8840e712839921 | 801 |
| 24cbb0da5567574041385f5d5b05ac77 | 799 |
| c08b14735cd16cae4fbfb9b636320639 | 797 |
| 807329b862e7576f521743d20c5ffd78 | 795 |
| 1425e15873d195069187835070016687 | 794 |
| 1b77cd6bd6e684e83e07b26af5a53677 | 792 |
| 77ffaf0d4c9c613a7d79eb2c4f621b4f | 792 |
| 8bb5ff097e0707dca3fe5860af20f4a6 | 792 |
| ac5402de1ddf427ab8d2b0a8a0a44f19 | 792 |
| f7686a74ca2d3729eb66305e8a26309b | 791 |
| 03c11654fe4e301be1c0979954621b09 | 789 |
| fe74365dbce3cf68d3f85a3a7d7b1750 | 789 |
| a7349158042db779f7c9734e0b320a88 | 787 |
| 413ea77358cd52ef4119be18a454b812 | 786 |
| 6234321dd90179936aa89249d23d256d | 786 |
| 2c6244626b7d94b8e4a351da9f2f661a | 785 |
| 60c57911662a9159dfdd0fc05d975a55 | 785 |
| 162c7ae1dc684b1769ffa348d920947b | 784 |
| 73c7bfcb93c50caeb0d5cbbf5d748b5c | 783 |
| f43f1f01f4bc79c75893eb5a62a52fef | 782 |
| 216ed7d400d264d649ca7f611d11402d | 782 |
| 6c7fa77831ae630967a6fbfc8ee47901 | 782 |
| 50760d56d87dc7961ff7955347f21d46 | 782 |
| 4d04d759f5a6615dac43060726239891 | 781 |
| eea6b86c0c75e740670ccc50613b1b23 | 781 |
| 335003e2c98aba3bd7e91022b14749d1 | 781 |
| e0e8356d65027a3fc1f6f9a76988f891 | 780 |
| 91dd7ea72afcd207e750dc2dd0242b88 | 779 |
| 992072d045462445dc239b35300af2cc | 778 |
| 78e2d7f265e221e0e3c1f24b25af17d4 | 778 |
| 9128dcf6c5d3e94600a2d146f5d866db | 777 |
| 9def8f6f951d15053c938a644f66125c | 777 |
| e6788cffb85a9e3ac4a3eac8d4f58630 | 777 |
| a9387e76477da8688775569cf540191b | 777 |
| ea3af29626a95b6c7b89d631bf1a739c | 777 |
| 90a685caa7eac21daa8a8f56df52eb29 | 776 |
| 879af2cc6bd04622e5ff03a9efa115db | 776 |
| 281ff55c72652f6631de4b83292c1657 | 776 |
| 96b52d7def1f78bf1d7cd7f77968d2a3 | 774 |
| 411c185537cc23097fed311d59233893 | 774 |
| e934683ea38718cc674e8c7f2694cf1b | 774 |
| a28739b246a86ed27ea0ce62abb73920 | 773 |
| 3958dd41c2306278a1352ca76c0d9ff3 | 773 |
| bd62e4581e5e1ae4e2b14ca04f2bff87 | 773 |
| 0a190c77db93f0e1dac85fe08561d4e8 | 773 |
| 54e443904e95d28ded3d1cf629adcf2e | 772 |
| dd7b5da3f418b5dd9a4629636201c6f4 | 772 |
| b32932df64f61391de895842e8df953f | 772 |
| f60cdc7aab497e4182b623913caf72ec | 772 |
| 583d53df0ef3b94496a5d47769126f3f | 770 |
| 94b000e59b7ac160b0416c9ab45ff0bf | 770 |
| 8cd5ffc8ace567b4a7587fef9b4d3647 | 770 |
| dcc848c112d2d53a870529268223fdbf | 770 |
| 39bb3fb5b2257fd2eacca408dc42c5d1 | 770 |
| 76fb5d96ac4d60581b56d0e4839db141 | 769 |
| bbc1c182c6e2efcf112d6e82da2d3280 | 769 |
| 7ac0356578578d4a2cc89eaaae4b2dbf | 769 |
| 7ddc9016a58ad40a548db51beb9d41b3 | 768 |
| 2a8a90cd5770804a5f23f425004de7e5 | 768 |
| b88e4cb83cff81f36f26c182e613ac61 | 768 |
| f3ce842957d12ee49c40e64b1173900d | 768 |
| 4d98afe170d0844f8886dba264563a46 | 768 |
| 705276e25ae9e2c3fd2511bb753981b1 | 768 |
| c8d26993af2ad4352c58cdc716fcc774 | 767 |
| 86c1d625be65befeb0a4e20e47a46cec | 767 |
| 74051291dca7519e45960f1a4a2c4a77 | 767 |
| 1f61b6a1c07889785ff4ab0e15f46ca5 | 766 |
| f4dbdbad28369592b5502b71865ad1d0 | 766 |
| 04c8be5a3a6ba2d70446812e99318905 | 766 |
| 524773d713cea2b4b6c239e1200ede69 | 766 |
| 0dd4b0666967bf9b871bd715856f4704 | 765 |
| 49abe7c6bc7d158b2ee34885fc0f8c66 | 765 |
| 87cc4e819670b67da655d68008dbba4f | 765 |
| e053921db232dfa54179aab54496f5e7 | 765 |
| 2f4a23ac0033e9857aa452fd6b524e9b | 765 |
| 490d2aec117bd7289577684ba3cc1b64 | 764 |
| eef60fa76e63f74b8cc3883e5997cfea | 764 |
| e857dec2d94e637e33e3df7435aaf6f8 | 764 |
| 012458b9050e020a433f1cb844082536 | 764 |
| 1f0ae738472d91009bcad0793ef226b2 | 764 |
| 63f210a3ba35855bc62771db3a8b4d0b | 764 |
| f9b52205cb2d03153fd394a290212a10 | 764 |
| e125250350eb41999febd079266ce58f | 763 |
| dadb874cf55015dada2a22cc32c1eda9 | 763 |
| 7225fa5df9583479ba76463ba7e7b058 | 762 |
| acaddcc70d590a9e203dd7607ae7f81a | 762 |
| 644c5814a1f5fa4d0db33b8876307981 | 762 |
| 995b272dbcd0c936016c988ea931b157 | 761 |
| 5fbaa1bbad4e8985376fb6e0ed7336bb | 761 |
| 6b3aa8d9a7ab78ee1993ff9359757d75 | 760 |
| ca9dec36ad0aa5a1473144ddcb74951d | 760 |
| 6e0adb64e054404f08fb1ccf2effd35f | 759 |
| 3699ce59bd5f8851d993fec7e15582f2 | 759 |
| b1f2146bef78066448576fceeb6b644b | 759 |
| 1b1555d2299b50b8b385efd1330bae0b | 758 |
| c4f9d95c604fd7328f4a931fc6c1a189 | 758 |
| b582cb10bc41a69e1b761b8e2d9bd6a0 | 757 |
| 9a98d72793fca56845304bb0b1e405df | 757 |
| 9fc888375a2ec9ad57f281ff5b0ec5da | 757 |
| 376917760e5aa918592416e980809536 | 757 |
| 458157e555a80f9ba01d9da37ef787eb | 756 |
| edc789f75b72793d2be0c0b1599121e4 | 755 |
| b9d6b7bdef86bc40fcc29b9636fbe9df | 755 |
| 85b932de09082a1141e0a18b95020115 | 754 |
| 6a17479edbe461431433bddb084f731e | 753 |
| a66dd8d31584930c2b0f811f572dddcf | 753 |
| d56128667cbee8137e636870bcd1520d | 753 |
| ac67195e9b147eabf4ae07aebccdb817 | 753 |
| af798ffc0b2345146af4b11177ebf92c | 752 |
| 1562897cb39a17c2e133d1e6714ae10b | 752 |
| 861aebcc774e48b7c4f769dbf99da4c3 | 751 |
| 1d567135f7dc7dd884fe9157642f9fa6 | 751 |
| 9a12d6feb273fb305b231be27b377a02 | 751 |
| 5324b49c8732221afba3ca0699e02fe1 | 750 |
| 6240207fd3edd24bf0799b5f427423a0 | 750 |
| 44936fbfe6d8bd401f0113a65e88c5a3 | 749 |
| 6d24fb8920d9d8c9862c743608af554e | 748 |
| c2a8f307c74b4885e947fe56949f6580 | 747 |
| 270d883b50531cb24cc2500092348caa | 746 |
| 83f45488b78a04989136fdfe184ec0e8 | 746 |
| f9f5f08e8da4cc9740518223846563db | 744 |
| 7b6c45ec622418432746c130f3e42345 | 742 |
| 0f92fcc184a951ff8b0914467ad2f8f9 | 742 |
| 502fdc648d6eac5cd6cf007ff357f737 | 742 |
| c53f173252f21234b0783ccc8fdb0add | 741 |
| 399bcf0feb12a20ca0731d8d65268dae | 740 |
| 305f77f140817cc55175d854c238e172 | 739 |
| 7a65b081641bfe6e58b936f3c745b1eb | 738 |
| a0fe714798fe5f4b80cb25e2bd8c5aad | 738 |

Download table as TSV

---

#### Percentile abundances of features by group

| Percentile | 0.0 | 25.0 | 50.0 | 75.0 | 100.0 | 0.0 | 25.0 | 50.0 | 75.0 | 100.0 |
| --- | --- | --- | --- | --- | --- | --- | --- | --- | --- | --- |
| Group | No | No | No | No | No | Yes | Yes | Yes | Yes | Yes |
| bda6b6e7da7508c68057140e7bd6cd8d | 1.0 | 1.0 | 1.0 | 1.0 | 5.0 | 26.0 | 319.25 | 923.0 | 1517.75 | 2262.0 |
| c64278b7c0c20b3412e0cf6b3bd546fb | 1.0 | 1.0 | 1.0 | 1.0 | 1.0 | 6.0 | 93.75 | 260.5 | 381.75 | 1627.0 |
| 9171cba1631b9a713a9d4c2e1c3408f0 | 1.0 | 1.0 | 1.0 | 1.0 | 1.0 | 1.0 | 51.00 | 283.5 | 994.75 | 2444.0 |
| ee2e7e1ec3775388a6a1000c5de4c483 | 1.0 | 1.0 | 1.0 | 1.0 | 2.0 | 1.0 | 89.25 | 203.0 | 387.25 | 1050.0 |
| 7ab1bc6052053650189083370055ae66 | 1.0 | 1.0 | 1.0 | 1.0 | 1.0 | 1.0 | 145.00 | 333.0 | 630.25 | 4055.0 |
| 3b092a59cd0e2d57483420cc994ec49c | 1.0 | 1.0 | 1.0 | 1.5 | 9.0 | 1.0 | 61.75 | 459.5 | 739.50 | 10180.0 |
| b1a153ff5a21342991138e7f7dd2658e | 1.0 | 1.0 | 1.0 | 1.0 | 4.0 | 1.0 | 59.25 | 152.5 | 424.50 | 532.0 |
| 0fb0f66b7e335cc4480b4f827e958ab3 | 1.0 | 1.0 | 1.0 | 1.0 | 4.0 | 1.0 | 46.75 | 178.0 | 420.25 | 1705.0 |
| d061a99148d649c4eca4836c5f4b5b68 | 1.0 | 1.0 | 1.0 | 1.0 | 4.0 | 1.0 | 98.25 | 157.0 | 322.00 | 1122.0 |
| 0ac7d4cacbb81c9c8182426c988a1c21 | 1.0 | 1.0 | 1.0 | 1.0 | 4.0 | 1.0 | 118.00 | 317.0 | 975.00 | 3439.0 |
| 7b0c4c30e3e9348105221123952bf547 | 1.0 | 1.0 | 1.0 | 1.0 | 3.0 | 11.0 | 33.75 | 123.0 | 324.25 | 954.0 |
| 8c8c1f03f8c009f91e286e431aa80623 | 1.0 | 1.0 | 1.0 | 1.0 | 3.0 | 1.0 | 84.25 | 280.0 | 522.00 | 1711.0 |
| 07d05a9cba0df11f278b17a0ea24eed3 | 1.0 | 1.0 | 1.0 | 3.0 | 6.0 | 1.0 | 92.00 | 204.5 | 933.50 | 3145.0 |
| 2ca99e2c6c0fcb152b4919fa00c4eeed | 1.0 | 1.0 | 1.0 | 1.0 | 4.0 | 1.0 | 102.25 | 185.5 | 338.75 | 1291.0 |
| 9ee8e39c88b826bd1bfc99589655f49d | 1.0 | 413.5 | 595.0 | 916.0 | 4423.0 | 1.0 | 1.00 | 1.0 | 1.50 | 4.0 |
| 3b17bc4edbddbe11a9a573e5eebf8d66 | 1.0 | 1.0 | 1.0 | 1.0 | 7.0 | 1.0 | 25.25 | 179.0 | 1507.75 | 8574.0 |
| 9dd9cefe38347b087dd318d82962cc03 | 1.0 | 1.0 | 1.0 | 1.0 | 1.0 | 1.0 | 32.25 | 135.5 | 243.00 | 429.0 |
| b696a6a2ff1f6de34e946662a88c6a24 | 1.0 | 1.0 | 1.0 | 1.0 | 1.0 | 1.0 | 63.00 | 104.0 | 179.75 | 1089.0 |
| e18e57dcbb611101406f8fb1d180b8a1 | 1.0 | 1.0 | 1.0 | 2.0 | 7.0 | 1.0 | 82.50 | 306.5 | 1688.75 | 6454.0 |
| b902443fbf36d9390d212daef4f9bb11 | 21.0 | 153.0 | 261.0 | 345.0 | 5244.0 | 1.0 | 1.00 | 1.0 | 1.00 | 1.0 |
| b89075e3356afd5634655288c5a3d1b8 | 1.0 | 1.0 | 1.0 | 1.0 | 4.0 | 1.0 | 41.50 | 457.5 | 819.00 | 1479.0 |
| 7de0b23e91fd1db3bfb5b37cb92936ae | 38.0 | 630.5 | 1298.0 | 2513.0 | 11778.0 | 1.0 | 3.75 | 7.0 | 9.25 | 11.0 |
| 1a494aa43d60de2b0130682daa0c8dd1 | 1.0 | 1.0 | 1.0 | 1.0 | 1.0 | 1.0 | 38.75 | 75.5 | 140.00 | 1858.0 |
| 2c5c5d710e1b9229efa72f98ca7b4e71 | 1.0 | 1.0 | 1.0 | 1.0 | 3.0 | 1.0 | 59.75 | 95.0 | 177.25 | 322.0 |
| 8ebe701cf6fe9fecac2a0d2ba0f74884 | 4.0 | 241.0 | 796.0 | 2382.5 | 7812.0 | 1.0 | 1.00 | 2.0 | 4.00 | 5.0 |
| 14988fea81d67867717b050e6b892e63 | 1.0 | 1.0 | 1.0 | 1.0 | 1.0 | 1.0 | 29.25 | 63.0 | 169.00 | 564.0 |
| b423592b0ae631882095792b27729b9c | 1.0 | 1.0 | 1.0 | 1.0 | 3.0 | 1.0 | 29.50 | 88.5 | 142.00 | 329.0 |
| cd688ebb10111cd2be59c7eb5fed07a3 | 1.0 | 1.0 | 1.0 | 1.0 | 174.0 | 6.0 | 33.00 | 124.0 | 240.25 | 944.0 |
| e6924b794a53c647f1a9509519f37c9a | 1.0 | 1.0 | 1.0 | 1.0 | 1.0 | 3.0 | 37.00 | 83.0 | 92.50 | 249.0 |
| 599ba6458e91e2527f358f547ea39261 | 1.0 | 1.0 | 1.0 | 1.0 | 2.0 | 1.0 | 45.25 | 61.0 | 92.50 | 530.0 |
| ac06b2ac668f171362228caae9c12747 | 1.0 | 1.0 | 1.0 | 1.0 | 1.0 | 1.0 | 28.25 | 43.5 | 197.50 | 447.0 |
| 08dd9f8036267148e2dc831fbb594689 | 28.0 | 90.0 | 183.0 | 345.0 | 955.0 | 1.0 | 1.00 | 1.0 | 1.00 | 1.0 |
| beef5a54aaee13f217ecede427cb3071 | 1.0 | 1.0 | 1.0 | 1.0 | 3.0 | 1.0 | 29.75 | 76.5 | 133.75 | 552.0 |
| 9c9210d344e78f933f4aa6da483f6683 | 1.0 | 1.0 | 1.0 | 1.0 | 1.0 | 1.0 | 31.00 | 271.0 | 763.25 | 1929.0 |
| 6436532cd975b8c8000f486deac9fb51 | 1.0 | 1.0 | 1.0 | 1.0 | 1.0 | 1.0 | 38.00 | 79.5 | 206.25 | 335.0 |
| e072125a988d90ca4d1c96a5ef091210 | 1.0 | 1.0 | 1.0 | 1.0 | 3.0 | 1.0 | 44.50 | 68.0 | 139.25 | 430.0 |
| e91f1c5b67ffecb5ca3ca3b393a50f00 | 1.0 | 1.0 | 1.0 | 1.0 | 2.0 | 1.0 | 12.00 | 90.0 | 460.00 | 1062.0 |
| 964f4677a8ea31cce60d46326065323b | 1.0 | 1.0 | 1.0 | 1.0 | 11.0 | 1.0 | 38.25 | 93.5 | 677.25 | 5357.0 |
| ecaa9f4e15a28f212d9bd60f749191ab | 1.0 | 1.0 | 1.0 | 1.0 | 1.0 | 1.0 | 26.00 | 69.0 | 256.25 | 1002.0 |
| c79b3d87e705bf6859d8b3a8a57577f9 | 1.0 | 1.0 | 1.0 | 1.0 | 3.0 | 1.0 | 93.25 | 346.0 | 543.25 | 1521.0 |
| 34584166f11d66d5f290bc19bc44edc4 | 1.0 | 1.0 | 1.0 | 1.0 | 3.0 | 1.0 | 30.75 | 82.5 | 379.00 | 760.0 |
| 957902900b34c7f1ec534fbd5c29b77a | 1.0 | 1.0 | 1.0 | 1.0 | 10.0 | 4.0 | 42.75 | 52.5 | 125.25 | 227.0 |
| 3996fdc07a002f768922b79975cae51c | 1.0 | 1.0 | 1.0 | 1.0 | 3.0 | 1.0 | 10.75 | 75.0 | 587.00 | 1371.0 |
| 6c36809e5d98ab855c5ef2b8534596d3 | 1.0 | 1.0 | 1.0 | 2.0 | 233.0 | 4.0 | 33.75 | 79.5 | 409.75 | 4002.0 |
| 8743c146ab4737302c555e0b4f96f710 | 1.0 | 1.0 | 1.0 | 1.0 | 1.0 | 1.0 | 42.50 | 51.0 | 112.50 | 645.0 |
| 56758836e2fc766051d575e08978cdec | 1.0 | 140.0 | 389.0 | 773.5 | 4551.0 | 1.0 | 1.00 | 1.0 | 1.00 | 1.0 |
| b0645cbec7653c887cb868a248d95874 | 1.0 | 1.0 | 1.0 | 1.0 | 1.0 | 1.0 | 17.00 | 78.0 | 180.75 | 484.0 |
| b70073c1864e1f977594ed75f642df15 | 1.0 | 1.0 | 1.0 | 1.0 | 4.0 | 1.0 | 19.50 | 60.5 | 133.25 | 417.0 |
| 0021d135d4ac12982cc8abdf2b38e23f | 1.0 | 1.0 | 1.0 | 1.0 | 1.0 | 1.0 | 14.50 | 129.5 | 428.00 | 697.0 |
| 476ee1e58388dfab6f1796baab4dc203 | 1.0 | 1.0 | 1.0 | 1.0 | 6.0 | 1.0 | 38.50 | 184.0 | 1086.25 | 4286.0 |
| e11cb2911aeaeb30eb0041b063506871 | 68.0 | 198.5 | 373.0 | 631.5 | 1480.0 | 1.0 | 1.00 | 1.0 | 2.25 | 553.0 |
| 7f8b90a32aa7cec1f34061cad0adc662 | 1.0 | 1.0 | 1.0 | 1.0 | 12.0 | 1.0 | 10.75 | 248.0 | 580.25 | 2125.0 |
| cae14798faeabb8e1b8840e712839921 | 1.0 | 1.0 | 1.0 | 1.0 | 4.0 | 5.0 | 16.00 | 37.0 | 62.00 | 156.0 |
| 24cbb0da5567574041385f5d5b05ac77 | 1.0 | 1.0 | 1.0 | 1.0 | 1.0 | 1.0 | 10.25 | 52.5 | 66.25 | 380.0 |
| c08b14735cd16cae4fbfb9b636320639 | 125.0 | 930.5 | 1706.0 | 3145.5 | 6566.0 | 1.0 | 3.00 | 4.5 | 7.00 | 4100.0 |
| 807329b862e7576f521743d20c5ffd78 | 1.0 | 1.0 | 1.0 | 1.0 | 132.0 | 14.0 | 41.25 | 62.5 | 172.00 | 1327.0 |
| 1425e15873d195069187835070016687 | 1.0 | 96.0 | 186.0 | 405.5 | 2972.0 | 1.0 | 1.00 | 1.0 | 1.00 | 1.0 |
| 1b77cd6bd6e684e83e07b26af5a53677 | 1.0 | 1.0 | 1.0 | 1.0 | 3.0 | 1.0 | 16.75 | 53.5 | 256.50 | 872.0 |
| 77ffaf0d4c9c613a7d79eb2c4f621b4f | 1.0 | 1.0 | 1.0 | 1.0 | 2.0 | 1.0 | 16.75 | 29.5 | 70.25 | 159.0 |
| 8bb5ff097e0707dca3fe5860af20f4a6 | 1.0 | 1.0 | 1.0 | 1.0 | 3.0 | 1.0 | 22.25 | 46.5 | 127.00 | 1419.0 |
| ac5402de1ddf427ab8d2b0a8a0a44f19 | 1.0 | 1.0 | 1.0 | 1.0 | 3.0 | 1.0 | 21.25 | 45.5 | 136.25 | 456.0 |
| f7686a74ca2d3729eb66305e8a26309b | 1.0 | 1.0 | 1.0 | 1.0 | 3.0 | 4.0 | 13.75 | 46.0 | 85.50 | 264.0 |
| 03c11654fe4e301be1c0979954621b09 | 1.0 | 1.0 | 1.0 | 1.0 | 1.0 | 1.0 | 15.75 | 27.5 | 56.50 | 479.0 |
| fe74365dbce3cf68d3f85a3a7d7b1750 | 1.0 | 1.0 | 1.0 | 1.0 | 1.0 | 1.0 | 1.75 | 181.0 | 443.25 | 2201.0 |
| a7349158042db779f7c9734e0b320a88 | 1.0 | 1.0 | 1.0 | 1.0 | 3.0 | 1.0 | 9.25 | 119.0 | 303.00 | 4625.0 |
| 413ea77358cd52ef4119be18a454b812 | 1.0 | 1.0 | 1.0 | 1.0 | 1.0 | 1.0 | 9.50 | 24.0 | 96.50 | 148.0 |
| 6234321dd90179936aa89249d23d256d | 1.0 | 899.0 | 2323.0 | 3392.5 | 13994.0 | 1.0 | 2.50 | 4.0 | 7.50 | 9697.0 |
| 2c6244626b7d94b8e4a351da9f2f661a | 1.0 | 1.0 | 3.0 | 4.0 | 10.0 | 4.0 | 13.50 | 38.0 | 70.75 | 99.0 |
| 60c57911662a9159dfdd0fc05d975a55 | 1.0 | 1.0 | 1.0 | 1.0 | 3.0 | 1.0 | 7.75 | 51.0 | 98.75 | 368.0 |
| 162c7ae1dc684b1769ffa348d920947b | 1.0 | 1.0 | 1.0 | 1.0 | 2.0 | 1.0 | 24.25 | 75.0 | 129.50 | 1752.0 |
| 73c7bfcb93c50caeb0d5cbbf5d748b5c | 1.0 | 1.0 | 1.0 | 1.0 | 1.0 | 1.0 | 6.75 | 59.0 | 150.50 | 237.0 |
| f43f1f01f4bc79c75893eb5a62a52fef | 1.0 | 1.0 | 1.0 | 1.0 | 4.0 | 1.0 | 25.00 | 120.0 | 254.25 | 1152.0 |
| 216ed7d400d264d649ca7f611d11402d | 1.0 | 1.0 | 1.0 | 1.0 | 1.0 | 1.0 | 13.50 | 33.5 | 69.25 | 230.0 |
| 6c7fa77831ae630967a6fbfc8ee47901 | 1.0 | 1.0 | 1.0 | 1.0 | 1.0 | 1.0 | 6.50 | 21.0 | 58.50 | 364.0 |
| 50760d56d87dc7961ff7955347f21d46 | 1.0 | 1.0 | 1.0 | 1.0 | 1.0 | 1.0 | 4.00 | 18.0 | 66.75 | 225.0 |
| 4d04d759f5a6615dac43060726239891 | 1.0 | 1.0 | 1.0 | 1.0 | 1.0 | 1.0 | 5.25 | 18.0 | 97.25 | 227.0 |
| eea6b86c0c75e740670ccc50613b1b23 | 1.0 | 1.0 | 1.0 | 1.0 | 1.0 | 1.0 | 10.25 | 35.0 | 96.25 | 352.0 |
| 335003e2c98aba3bd7e91022b14749d1 | 1.0 | 1.0 | 1.0 | 1.0 | 5.0 | 1.0 | 1.00 | 289.0 | 1168.75 | 2389.0 |
| e0e8356d65027a3fc1f6f9a76988f891 | 1.0 | 1.0 | 1.0 | 1.0 | 1.0 | 1.0 | 6.25 | 39.0 | 78.75 | 221.0 |
| 91dd7ea72afcd207e750dc2dd0242b88 | 1.0 | 1.0 | 1.0 | 1.0 | 1.0 | 1.0 | 3.75 | 12.0 | 66.00 | 564.0 |
| 992072d045462445dc239b35300af2cc | 1.0 | 1.0 | 1.0 | 1.0 | 3.0 | 1.0 | 8.75 | 31.5 | 77.25 | 275.0 |
| 78e2d7f265e221e0e3c1f24b25af17d4 | 1.0 | 1.0 | 1.0 | 1.0 | 1.0 | 1.0 | 4.75 | 18.5 | 53.75 | 238.0 |
| 9128dcf6c5d3e94600a2d146f5d866db | 1.0 | 1.0 | 1.0 | 1.0 | 81.0 | 1.0 | 22.25 | 29.5 | 78.75 | 153.0 |
| 9def8f6f951d15053c938a644f66125c | 1.0 | 1.0 | 1.0 | 1.0 | 1.0 | 1.0 | 9.75 | 54.5 | 94.50 | 342.0 |
| e6788cffb85a9e3ac4a3eac8d4f58630 | 1.0 | 1.0 | 1.0 | 1.0 | 1.0 | 1.0 | 4.75 | 19.5 | 58.75 | 192.0 |
| a9387e76477da8688775569cf540191b | 1.0 | 1.0 | 1.0 | 2.5 | 46.0 | 4.0 | 19.75 | 112.0 | 144.50 | 1456.0 |
| ea3af29626a95b6c7b89d631bf1a739c | 1.0 | 1.0 | 1.0 | 1.0 | 1.0 | 1.0 | 1.00 | 16.5 | 29.00 | 373.0 |
| 90a685caa7eac21daa8a8f56df52eb29 | 1.0 | 1.0 | 1.0 | 1.0 | 3.0 | 1.0 | 4.00 | 28.5 | 76.50 | 402.0 |
| 879af2cc6bd04622e5ff03a9efa115db | 1.0 | 1.0 | 1.0 | 1.0 | 1.0 | 1.0 | 5.50 | 14.0 | 64.00 | 276.0 |
| 281ff55c72652f6631de4b83292c1657 | 1.0 | 1.0 | 1.0 | 1.0 | 1.0 | 1.0 | 3.75 | 33.0 | 156.75 | 291.0 |
| 96b52d7def1f78bf1d7cd7f77968d2a3 | 1.0 | 1.0 | 1.0 | 1.0 | 4.0 | 1.0 | 6.00 | 33.0 | 91.75 | 559.0 |
| 411c185537cc23097fed311d59233893 | 1.0 | 1.0 | 1.0 | 1.0 | 1.0 | 1.0 | 8.50 | 17.5 | 45.25 | 242.0 |
| e934683ea38718cc674e8c7f2694cf1b | 1.0 | 1.0 | 1.0 | 1.0 | 1.0 | 1.0 | 3.75 | 20.0 | 33.50 | 354.0 |
| a28739b246a86ed27ea0ce62abb73920 | 1.0 | 1.0 | 1.0 | 1.0 | 2.0 | 1.0 | 1.00 | 3.0 | 16.75 | 256.0 |
| 3958dd41c2306278a1352ca76c0d9ff3 | 1.0 | 1.0 | 1.0 | 1.0 | 1.0 | 1.0 | 3.75 | 23.5 | 48.75 | 1005.0 |
| bd62e4581e5e1ae4e2b14ca04f2bff87 | 1.0 | 1.0 | 1.0 | 1.0 | 1.0 | 1.0 | 1.00 | 40.0 | 128.25 | 501.0 |
| 0a190c77db93f0e1dac85fe08561d4e8 | 1.0 | 1.0 | 1.0 | 1.0 | 1.0 | 1.0 | 6.25 | 33.0 | 70.25 | 640.0 |
| 54e443904e95d28ded3d1cf629adcf2e | 1.0 | 1.0 | 1.0 | 1.0 | 1.0 | 1.0 | 8.00 | 26.0 | 74.50 | 276.0 |
| dd7b5da3f418b5dd9a4629636201c6f4 | 1.0 | 1.0 | 1.0 | 1.0 | 2.0 | 1.0 | 1.00 | 146.0 | 545.75 | 1567.0 |
| b32932df64f61391de895842e8df953f | 1.0 | 1.0 | 1.0 | 1.0 | 1.0 | 1.0 | 1.00 | 8.0 | 84.25 | 403.0 |
| f60cdc7aab497e4182b623913caf72ec | 1.0 | 1.0 | 1.0 | 1.0 | 3.0 | 1.0 | 2.50 | 117.5 | 388.00 | 1170.0 |
| 583d53df0ef3b94496a5d47769126f3f | 1.0 | 1.0 | 1.0 | 1.0 | 1.0 | 1.0 | 1.00 | 63.0 | 212.75 | 298.0 |
| 94b000e59b7ac160b0416c9ab45ff0bf | 3.0 | 5.0 | 7.0 | 13.0 | 105.0 | 5.0 | 35.75 | 100.0 | 417.50 | 1292.0 |
| 8cd5ffc8ace567b4a7587fef9b4d3647 | 1.0 | 1.0 | 1.0 | 1.0 | 1.0 | 1.0 | 8.50 | 39.5 | 122.00 | 503.0 |
| dcc848c112d2d53a870529268223fdbf | 1.0 | 1.0 | 1.0 | 1.0 | 2.0 | 1.0 | 1.00 | 36.0 | 72.75 | 213.0 |
| 39bb3fb5b2257fd2eacca408dc42c5d1 | 1.0 | 1.0 | 1.0 | 1.0 | 1.0 | 1.0 | 6.25 | 21.5 | 92.25 | 362.0 |
| 76fb5d96ac4d60581b56d0e4839db141 | 1.0 | 1.0 | 1.0 | 1.0 | 2.0 | 1.0 | 6.75 | 14.5 | 39.75 | 367.0 |
| bbc1c182c6e2efcf112d6e82da2d3280 | 1.0 | 1.0 | 1.0 | 1.0 | 1.0 | 1.0 | 1.00 | 51.5 | 147.25 | 260.0 |
| 7ac0356578578d4a2cc89eaaae4b2dbf | 1.0 | 1.0 | 1.0 | 1.0 | 4.0 | 1.0 | 4.00 | 45.0 | 123.50 | 3382.0 |
| 7ddc9016a58ad40a548db51beb9d41b3 | 1.0 | 1.0 | 1.0 | 1.0 | 1.0 | 1.0 | 1.00 | 82.0 | 139.25 | 1069.0 |
| 2a8a90cd5770804a5f23f425004de7e5 | 1.0 | 1.0 | 1.0 | 1.0 | 1.0 | 1.0 | 4.00 | 19.0 | 64.00 | 138.0 |
| b88e4cb83cff81f36f26c182e613ac61 | 1.0 | 1.0 | 1.0 | 1.0 | 1.0 | 1.0 | 1.00 | 29.5 | 104.75 | 282.0 |
| f3ce842957d12ee49c40e64b1173900d | 1.0 | 1.0 | 1.0 | 1.0 | 3.0 | 1.0 | 1.00 | 77.0 | 663.50 | 1244.0 |
| 4d98afe170d0844f8886dba264563a46 | 1.0 | 1.0 | 1.0 | 1.0 | 1.0 | 1.0 | 1.00 | 9.0 | 38.00 | 448.0 |
| 705276e25ae9e2c3fd2511bb753981b1 | 1.0 | 1.0 | 1.0 | 1.0 | 1.0 | 1.0 | 1.75 | 18.0 | 83.25 | 250.0 |
| c8d26993af2ad4352c58cdc716fcc774 | 1.0 | 1.0 | 1.0 | 1.0 | 16.0 | 1.0 | 1.00 | 15.0 | 79.75 | 1147.0 |
| 86c1d625be65befeb0a4e20e47a46cec | 1.0 | 1.0 | 1.0 | 1.0 | 3.0 | 1.0 | 4.50 | 21.5 | 108.00 | 2071.0 |
| 74051291dca7519e45960f1a4a2c4a77 | 1.0 | 1.0 | 1.0 | 1.0 | 1.0 | 1.0 | 1.00 | 84.0 | 281.75 | 1689.0 |
| 1f61b6a1c07889785ff4ab0e15f46ca5 | 1.0 | 1.0 | 1.0 | 1.0 | 1.0 | 1.0 | 3.25 | 26.0 | 71.00 | 353.0 |
| f4dbdbad28369592b5502b71865ad1d0 | 1.0 | 1.0 | 1.0 | 1.0 | 1.0 | 1.0 | 11.00 | 19.5 | 50.50 | 771.0 |
| 04c8be5a3a6ba2d70446812e99318905 | 1.0 | 1.0 | 1.0 | 3.0 | 264.0 | 1.0 | 29.00 | 75.5 | 179.75 | 1593.0 |
| 524773d713cea2b4b6c239e1200ede69 | 12.0 | 76.0 | 131.0 | 221.0 | 1769.0 | 1.0 | 1.00 | 1.0 | 1.00 | 3.0 |
| 0dd4b0666967bf9b871bd715856f4704 | 1.0 | 1.0 | 1.0 | 2.0 | 4.0 | 1.0 | 4.75 | 27.0 | 378.50 | 2416.0 |
| 49abe7c6bc7d158b2ee34885fc0f8c66 | 1.0 | 1.0 | 1.0 | 1.0 | 3.0 | 1.0 | 2.50 | 38.0 | 144.25 | 532.0 |
| 87cc4e819670b67da655d68008dbba4f | 1.0 | 1.0 | 1.0 | 1.0 | 4.0 | 1.0 | 1.00 | 139.0 | 1136.75 | 3443.0 |
| e053921db232dfa54179aab54496f5e7 | 1.0 | 1.0 | 1.0 | 1.0 | 1.0 | 1.0 | 2.50 | 37.0 | 136.50 | 1233.0 |
| 2f4a23ac0033e9857aa452fd6b524e9b | 1.0 | 1.0 | 1.0 | 1.0 | 1.0 | 1.0 | 1.00 | 60.0 | 175.25 | 738.0 |
| 490d2aec117bd7289577684ba3cc1b64 | 1.0 | 1.0 | 1.0 | 1.0 | 3.0 | 1.0 | 4.00 | 58.0 | 165.75 | 263.0 |
| eef60fa76e63f74b8cc3883e5997cfea | 1.0 | 1.0 | 1.0 | 1.0 | 3.0 | 1.0 | 1.00 | 100.0 | 179.00 | 989.0 |
| e857dec2d94e637e33e3df7435aaf6f8 | 1.0 | 1.0 | 1.0 | 1.0 | 1.0 | 1.0 | 1.00 | 41.0 | 74.75 | 596.0 |
| 012458b9050e020a433f1cb844082536 | 1.0 | 1.0 | 1.0 | 1.0 | 1.0 | 1.0 | 6.25 | 17.0 | 46.00 | 247.0 |
| 1f0ae738472d91009bcad0793ef226b2 | 1.0 | 1.0 | 1.0 | 1.0 | 1.0 | 1.0 | 1.00 | 47.0 | 452.25 | 680.0 |
| 63f210a3ba35855bc62771db3a8b4d0b | 1.0 | 1.0 | 1.0 | 1.0 | 5.0 | 1.0 | 1.00 | 13.0 | 166.75 | 331.0 |
| f9b52205cb2d03153fd394a290212a10 | 1.0 | 1.0 | 1.0 | 1.0 | 1.0 | 1.0 | 2.50 | 60.5 | 89.00 | 414.0 |
| e125250350eb41999febd079266ce58f | 1.0 | 1.0 | 1.0 | 1.0 | 6.0 | 1.0 | 1.00 | 258.0 | 1048.25 | 2025.0 |
| dadb874cf55015dada2a22cc32c1eda9 | 1.0 | 714.5 | 2270.0 | 5557.5 | 23843.0 | 3.0 | 3.75 | 5.5 | 8.75 | 13.0 |
| 7225fa5df9583479ba76463ba7e7b058 | 1.0 | 1.0 | 1.0 | 1.0 | 20.0 | 1.0 | 7.75 | 29.5 | 88.00 | 287.0 |
| acaddcc70d590a9e203dd7607ae7f81a | 1.0 | 74.5 | 121.0 | 227.5 | 938.0 | 1.0 | 1.00 | 1.0 | 1.00 | 1.0 |
| 644c5814a1f5fa4d0db33b8876307981 | 1.0 | 1.0 | 1.0 | 1.0 | 1.0 | 1.0 | 1.00 | 60.0 | 137.25 | 387.0 |
| 995b272dbcd0c936016c988ea931b157 | 1.0 | 1.0 | 1.0 | 1.0 | 1.0 | 1.0 | 1.00 | 17.5 | 70.25 | 243.0 |
| 5fbaa1bbad4e8985376fb6e0ed7336bb | 10.0 | 59.0 | 102.0 | 151.5 | 1301.0 | 1.0 | 1.00 | 1.0 | 1.00 | 1.0 |
| 6b3aa8d9a7ab78ee1993ff9359757d75 | 1.0 | 1.0 | 1.0 | 1.0 | 1.0 | 1.0 | 1.00 | 12.5 | 26.25 | 559.0 |
| ca9dec36ad0aa5a1473144ddcb74951d | 1.0 | 1.0 | 1.0 | 1.0 | 1.0 | 1.0 | 1.75 | 16.5 | 76.25 | 280.0 |
| 6e0adb64e054404f08fb1ccf2effd35f | 1.0 | 1.0 | 1.0 | 1.0 | 1.0 | 1.0 | 1.00 | 10.0 | 29.25 | 576.0 |
| 3699ce59bd5f8851d993fec7e15582f2 | 1.0 | 1.0 | 1.0 | 1.0 | 1.0 | 1.0 | 1.00 | 8.5 | 80.25 | 780.0 |
| b1f2146bef78066448576fceeb6b644b | 1.0 | 1.0 | 1.0 | 1.0 | 4.0 | 1.0 | 1.00 | 31.5 | 147.25 | 1564.0 |
| 1b1555d2299b50b8b385efd1330bae0b | 1.0 | 1.0 | 1.0 | 1.0 | 3.0 | 1.0 | 1.00 | 22.5 | 51.75 | 695.0 |
| c4f9d95c604fd7328f4a931fc6c1a189 | 1.0 | 1.0 | 1.0 | 1.0 | 41.0 | 1.0 | 8.75 | 29.0 | 93.50 | 360.0 |
| b582cb10bc41a69e1b761b8e2d9bd6a0 | 1.0 | 1.0 | 1.0 | 1.0 | 429.0 | 5.0 | 15.25 | 29.5 | 150.00 | 635.0 |
| 9a98d72793fca56845304bb0b1e405df | 1.0 | 1.0 | 1.0 | 1.0 | 1.0 | 1.0 | 1.00 | 59.0 | 133.50 | 275.0 |
| 9fc888375a2ec9ad57f281ff5b0ec5da | 1.0 | 1.0 | 1.0 | 1.0 | 2.0 | 1.0 | 1.00 | 35.5 | 87.75 | 358.0 |
| 376917760e5aa918592416e980809536 | 1.0 | 1.0 | 1.0 | 1.0 | 4.0 | 1.0 | 1.75 | 53.5 | 189.25 | 10428.0 |
| 458157e555a80f9ba01d9da37ef787eb | 1.0 | 1.0 | 1.0 | 1.0 | 2.0 | 1.0 | 1.00 | 65.0 | 139.00 | 1232.0 |
| edc789f75b72793d2be0c0b1599121e4 | 1.0 | 1.0 | 1.0 | 1.0 | 48.0 | 1.0 | 7.00 | 49.0 | 174.75 | 605.0 |
| b9d6b7bdef86bc40fcc29b9636fbe9df | 1.0 | 1.0 | 1.0 | 1.0 | 2.0 | 1.0 | 1.00 | 16.5 | 308.00 | 5701.0 |
| 85b932de09082a1141e0a18b95020115 | 1.0 | 1.0 | 1.0 | 1.0 | 32.0 | 1.0 | 2.50 | 21.5 | 139.75 | 649.0 |
| 6a17479edbe461431433bddb084f731e | 1.0 | 1.0 | 1.0 | 1.0 | 3.0 | 1.0 | 1.00 | 6.0 | 36.00 | 1416.0 |
| a66dd8d31584930c2b0f811f572dddcf | 1.0 | 1.0 | 1.0 | 5.0 | 29.0 | 1.0 | 29.25 | 72.0 | 192.75 | 1546.0 |
| d56128667cbee8137e636870bcd1520d | 1.0 | 1.0 | 1.0 | 1.0 | 3.0 | 1.0 | 1.00 | 20.0 | 403.25 | 1308.0 |
| ac67195e9b147eabf4ae07aebccdb817 | 1.0 | 1.0 | 1.0 | 1.0 | 1.0 | 1.0 | 1.00 | 19.5 | 104.75 | 762.0 |
| af798ffc0b2345146af4b11177ebf92c | 1.0 | 65.0 | 122.0 | 216.0 | 1053.0 | 1.0 | 1.00 | 1.0 | 1.00 | 4.0 |
| 1562897cb39a17c2e133d1e6714ae10b | 1.0 | 1.0 | 1.0 | 1.0 | 1.0 | 1.0 | 1.00 | 35.5 | 235.50 | 973.0 |
| 861aebcc774e48b7c4f769dbf99da4c3 | 1.0 | 1.0 | 1.0 | 1.0 | 1.0 | 1.0 | 1.00 | 32.0 | 95.50 | 1363.0 |
| 1d567135f7dc7dd884fe9157642f9fa6 | 1.0 | 1.0 | 1.0 | 2.5 | 119.0 | 1.0 | 8.50 | 317.5 | 1442.25 | 3628.0 |
| 9a12d6feb273fb305b231be27b377a02 | 1.0 | 1.0 | 1.0 | 3.0 | 5.0 | 1.0 | 1.00 | 42.0 | 2498.25 | 13593.0 |
| 5324b49c8732221afba3ca0699e02fe1 | 1.0 | 1.0 | 1.0 | 1.0 | 4.0 | 1.0 | 1.00 | 30.0 | 381.25 | 8467.0 |
| 6240207fd3edd24bf0799b5f427423a0 | 1.0 | 1.0 | 1.0 | 9.0 | 88.0 | 5.0 | 30.25 | 59.0 | 110.25 | 292.0 |
| 44936fbfe6d8bd401f0113a65e88c5a3 | 1.0 | 1.0 | 1.0 | 3.0 | 32.0 | 1.0 | 8.00 | 14.5 | 53.25 | 322.0 |
| 6d24fb8920d9d8c9862c743608af554e | 1.0 | 1.0 | 1.0 | 1.0 | 1.0 | 1.0 | 1.00 | 8.5 | 42.75 | 1483.0 |
| c2a8f307c74b4885e947fe56949f6580 | 1.0 | 1.0 | 1.0 | 1.0 | 5.0 | 1.0 | 1.00 | 3.0 | 152.75 | 1101.0 |
| 270d883b50531cb24cc2500092348caa | 1.0 | 1.0 | 1.0 | 1.0 | 3.0 | 1.0 | 1.00 | 28.0 | 332.25 | 1012.0 |
| 83f45488b78a04989136fdfe184ec0e8 | 1.0 | 158.0 | 279.0 | 539.0 | 2629.0 | 1.0 | 1.00 | 1.0 | 1.00 | 215.0 |
| f9f5f08e8da4cc9740518223846563db | 1.0 | 1.0 | 1.0 | 7.5 | 99.0 | 1.0 | 51.50 | 144.5 | 330.50 | 558.0 |
| 7b6c45ec622418432746c130f3e42345 | 1.0 | 1.0 | 1.0 | 1.0 | 3.0 | 1.0 | 3.25 | 15.0 | 47.75 | 1309.0 |
| 0f92fcc184a951ff8b0914467ad2f8f9 | 1.0 | 1.0 | 1.0 | 4.0 | 27.0 | 1.0 | 4.00 | 16.5 | 48.75 | 94.0 |
| 502fdc648d6eac5cd6cf007ff357f737 | 1.0 | 271.5 | 736.0 | 1485.0 | 7352.0 | 1.0 | 1.00 | 4.0 | 6.25 | 246.0 |
| c53f173252f21234b0783ccc8fdb0add | 1.0 | 1.0 | 3.0 | 6.0 | 109.0 | 1.0 | 16.25 | 44.5 | 82.00 | 263.0 |
| 399bcf0feb12a20ca0731d8d65268dae | 1.0 | 1.0 | 1.0 | 3.5 | 653.0 | 1.0 | 1.75 | 908.0 | 3019.00 | 8047.0 |
| 305f77f140817cc55175d854c238e172 | 1.0 | 1.0 | 1.0 | 1.0 | 14.0 | 1.0 | 1.00 | 28.0 | 138.75 | 472.0 |
| 7a65b081641bfe6e58b936f3c745b1eb | 1.0 | 74.5 | 179.0 | 313.0 | 862.0 | 1.0 | 1.00 | 1.0 | 1.00 | 3.0 |
| a0fe714798fe5f4b80cb25e2bd8c5aad | 1.0 | 1.0 | 1.0 | 1.0 | 3.0 | 1.0 | 1.00 | 3.5 | 127.00 | 1038.0 |

Download table as TSV
