# Supplementary material for: Signatures of landscape and captivity in the gut microbiota of Southern Hairy-nosed Wombats (Lasiorhinus latifrons)
Source: Anim Microbiome. 2021 Jan 6;3:4. doi: 10.1186/s42523-020-00068-y (PMC7934541; doi:10.1186/s42523-020-00068-y)
Supplement: Supplementary file 12 — Additional file 12: SI_File_4. QIIME2 qzv file of ANCOM test at family level (between different wild populations). [file 42523_2020_68_MOESM12_ESM.qzv › e9b87620-32b1-4026-9967-d36d474314c8/data/index.html]

q2\_composition : ancom


### ANCOM Volcano Plot

---

#### ANCOM statistical results

|  | W |
| --- | --- |
| D\_0\_\_Bacteria;D\_1\_\_Tenericutes;D\_2\_\_Mollicutes;D\_3\_\_Mollicutes RF39;D\_4\_\_gut metagenome | 91 |
| D\_0\_\_Bacteria;D\_1\_\_Firmicutes;D\_2\_\_Bacilli;D\_3\_\_Lactobacillales;D\_4\_\_Streptococcaceae | 89 |
| D\_0\_\_Bacteria;D\_1\_\_Actinobacteria;D\_2\_\_Actinobacteria;D\_3\_\_Micrococcales;D\_4\_\_Micrococcaceae | 87 |
| D\_0\_\_Bacteria;D\_1\_\_Tenericutes;D\_2\_\_Mollicutes;D\_3\_\_T2WK15B57;D\_4\_\_uncultured bacterium | 86 |
| D\_0\_\_Bacteria;D\_1\_\_Tenericutes;D\_2\_\_Mollicutes;D\_3\_\_Mollicutes RF39;D\_4\_\_uncultured rumen bacterium | 86 |
| D\_0\_\_Bacteria;D\_1\_\_Proteobacteria;D\_2\_\_Alphaproteobacteria;D\_3\_\_Rhodobacterales;D\_4\_\_Rhodobacteraceae | 85 |
| D\_0\_\_Bacteria;D\_1\_\_Actinobacteria;D\_2\_\_Actinobacteria;D\_3\_\_Frankiales;D\_4\_\_Geodermatophilaceae | 78 |

Download table as TSV

---

#### Percentile abundances of features by group

| Percentile | 0.0 | 25.0 | 50.0 | 75.0 | 100.0 | 0.0 | 25.0 | 50.0 | 75.0 | 100.0 | 0.0 | 25.0 | 50.0 | 75.0 | 100.0 |
| --- | --- | --- | --- | --- | --- | --- | --- | --- | --- | --- | --- | --- | --- | --- | --- |
| Group | Brookfield | Brookfield | Brookfield | Brookfield | Brookfield | Kooloola | Kooloola | Kooloola | Kooloola | Kooloola | Wonga | Wonga | Wonga | Wonga | Wonga |
| D\_0\_\_Bacteria;D\_1\_\_Tenericutes;D\_2\_\_Mollicutes;D\_3\_\_Mollicutes RF39;D\_4\_\_gut metagenome | 1.0 | 316.0 | 420.0 | 1022.0 | 3437.0 | 1.0 | 7.50 | 56.5 | 111.75 | 1630.0 | 1.0 | 1.00 | 1.0 | 1.00 | 1.0 |
| D\_0\_\_Bacteria;D\_1\_\_Firmicutes;D\_2\_\_Bacilli;D\_3\_\_Lactobacillales;D\_4\_\_Streptococcaceae | 8.0 | 16.0 | 18.0 | 37.0 | 443.0 | 20.0 | 221.75 | 499.0 | 1266.00 | 3875.0 | 11.0 | 28.75 | 54.0 | 125.25 | 443.0 |
| D\_0\_\_Bacteria;D\_1\_\_Actinobacteria;D\_2\_\_Actinobacteria;D\_3\_\_Micrococcales;D\_4\_\_Micrococcaceae | 1.0 | 4.0 | 7.0 | 9.0 | 18.0 | 1.0 | 1.00 | 2.0 | 5.50 | 192.0 | 41.0 | 123.50 | 160.5 | 299.00 | 2392.0 |
| D\_0\_\_Bacteria;D\_1\_\_Tenericutes;D\_2\_\_Mollicutes;D\_3\_\_T2WK15B57;D\_4\_\_uncultured bacterium | 1.0 | 1.0 | 1.0 | 1.0 | 1.0 | 1.0 | 1.00 | 13.0 | 49.00 | 417.0 | 1.0 | 1.00 | 1.0 | 1.00 | 1.0 |
| D\_0\_\_Bacteria;D\_1\_\_Tenericutes;D\_2\_\_Mollicutes;D\_3\_\_Mollicutes RF39;D\_4\_\_uncultured rumen bacterium | 1.0 | 36.0 | 94.0 | 161.0 | 306.0 | 1.0 | 1.00 | 1.0 | 1.00 | 53.0 | 1.0 | 1.00 | 1.0 | 10.50 | 60.0 |
| D\_0\_\_Bacteria;D\_1\_\_Proteobacteria;D\_2\_\_Alphaproteobacteria;D\_3\_\_Rhodobacterales;D\_4\_\_Rhodobacteraceae | 1.0 | 1.0 | 1.0 | 3.0 | 9.0 | 1.0 | 1.00 | 1.0 | 1.00 | 39.0 | 22.0 | 41.50 | 78.5 | 132.50 | 376.0 |
| D\_0\_\_Bacteria;D\_1\_\_Actinobacteria;D\_2\_\_Actinobacteria;D\_3\_\_Frankiales;D\_4\_\_Geodermatophilaceae | 1.0 | 1.0 | 4.0 | 4.0 | 13.0 | 1.0 | 1.00 | 4.0 | 7.25 | 19.0 | 8.0 | 57.50 | 86.0 | 113.75 | 200.0 |

Download table as TSV
