# Supplementary material for: Signatures of landscape and captivity in the gut microbiota of Southern Hairy-nosed Wombats (Lasiorhinus latifrons)
Source: Anim Microbiome. 2021 Jan 6;3:4. doi: 10.1186/s42523-020-00068-y (PMC7934541; doi:10.1186/s42523-020-00068-y)
Supplement: Supplementary file 13 — Additional file 13: SI_File_5. QIIME2 qzv file of ANCOM test at the ASV level (between different wild populations). [file 42523_2020_68_MOESM13_ESM.qzv › 4d21941b-7e47-4727-ba22-2776461520b3/data/index.html]

q2\_composition : ancom


### ANCOM Volcano Plot

---

#### ANCOM statistical results

|  | W |
| --- | --- |
| 45127f608cc1fcfeffc2026d2ade2c97 | 779 |
| 7a199378c70bfd0040603527b43f794d | 772 |
| 06ebc3bfac4d5e667890bef6da8f72c3 | 761 |
| b80972d9e16f3aa7666859b612399f66 | 759 |
| 350715558fc9870039b34bc1f1dff39f | 759 |
| d798f8dc15cd25304cd90385b3474bbb | 757 |
| 2420d39e9c3ca631e33ea9f330614698 | 740 |
| bf0f3ada36af7e7425a1704816ee743a | 740 |
| caacf902f6de815cd8b8a6016886dcd9 | 738 |
| 3e1a1bffd8f3c4c02c34162195f70585 | 734 |
| 08e700017fcf7b2701ed3ce0ccd3f3ae | 728 |
| 14a999ac116871041248e75a737e4362 | 727 |
| 34561f902bdd50d502f4dead584acf01 | 721 |
| fa2c3310c83195e117798d944f1e7f29 | 708 |

Download table as TSV

---

#### Percentile abundances of features by group

| Percentile | 0.0 | 25.0 | 50.0 | 75.0 | 100.0 | 0.0 | 25.0 | 50.0 | 75.0 | 100.0 | 0.0 | 25.0 | 50.0 | 75.0 | 100.0 |
| --- | --- | --- | --- | --- | --- | --- | --- | --- | --- | --- | --- | --- | --- | --- | --- |
| Group | Brookfield | Brookfield | Brookfield | Brookfield | Brookfield | Kooloola | Kooloola | Kooloola | Kooloola | Kooloola | Wonga | Wonga | Wonga | Wonga | Wonga |
| 45127f608cc1fcfeffc2026d2ade2c97 | 1.0 | 1.0 | 1.0 | 1.0 | 5.0 | 1.0 | 138.00 | 433.0 | 1197.50 | 3875.0 | 1.0 | 1.00 | 1.0 | 1.00 | 4.0 |
| 7a199378c70bfd0040603527b43f794d | 1.0 | 1.0 | 1.0 | 4.0 | 123.0 | 1.0 | 1.00 | 1.0 | 1.75 | 268.0 | 60.0 | 243.25 | 686.0 | 995.25 | 4623.0 |
| 06ebc3bfac4d5e667890bef6da8f72c3 | 1.0 | 1.0 | 1.0 | 1.0 | 2.0 | 1.0 | 1.00 | 1.0 | 1.00 | 21.0 | 1.0 | 90.75 | 263.0 | 606.50 | 5907.0 |
| b80972d9e16f3aa7666859b612399f66 | 1.0 | 14.0 | 21.0 | 29.0 | 53.0 | 10.0 | 55.75 | 122.5 | 191.25 | 307.0 | 1.0 | 1.00 | 1.0 | 1.00 | 1.0 |
| 350715558fc9870039b34bc1f1dff39f | 32.0 | 99.0 | 293.0 | 2103.0 | 2912.0 | 15.0 | 130.00 | 169.0 | 352.50 | 1218.0 | 1.0 | 1.00 | 1.0 | 3.00 | 7.0 |
| d798f8dc15cd25304cd90385b3474bbb | 43.0 | 76.0 | 138.0 | 270.0 | 378.0 | 1.0 | 3.75 | 20.0 | 86.00 | 163.0 | 402.0 | 1132.25 | 1993.5 | 2631.00 | 6111.0 |
| 2420d39e9c3ca631e33ea9f330614698 | 1.0 | 8.0 | 15.0 | 18.0 | 27.0 | 15.0 | 41.75 | 63.5 | 77.50 | 120.0 | 1.0 | 1.00 | 1.0 | 1.00 | 1.0 |
| bf0f3ada36af7e7425a1704816ee743a | 18.0 | 66.0 | 335.0 | 867.0 | 2078.0 | 1.0 | 1.00 | 1.0 | 3.00 | 77.0 | 1.0 | 1.00 | 1.0 | 1.00 | 1.0 |
| caacf902f6de815cd8b8a6016886dcd9 | 75.0 | 141.0 | 352.0 | 852.0 | 1613.0 | 38.0 | 160.00 | 210.5 | 393.00 | 936.0 | 1.0 | 1.00 | 1.0 | 7.00 | 70.0 |
| 3e1a1bffd8f3c4c02c34162195f70585 | 114.0 | 488.0 | 762.0 | 1669.0 | 4014.0 | 1.0 | 4.00 | 85.5 | 699.00 | 3433.0 | 1.0 | 1.00 | 1.0 | 1.00 | 5.0 |
| 08e700017fcf7b2701ed3ce0ccd3f3ae | 50.0 | 197.0 | 242.0 | 340.0 | 599.0 | 11.0 | 49.75 | 82.0 | 195.50 | 317.0 | 1.0 | 1.00 | 1.0 | 3.00 | 23.0 |
| 14a999ac116871041248e75a737e4362 | 8.0 | 150.0 | 193.0 | 492.0 | 590.0 | 1.0 | 151.25 | 272.0 | 1345.75 | 2234.0 | 1.0 | 1.00 | 1.0 | 5.50 | 126.0 |
| 34561f902bdd50d502f4dead584acf01 | 14.0 | 45.0 | 176.0 | 262.0 | 359.0 | 1.0 | 85.50 | 123.0 | 171.00 | 466.0 | 1.0 | 1.00 | 1.0 | 1.00 | 50.0 |
| fa2c3310c83195e117798d944f1e7f29 | 21.0 | 116.0 | 227.0 | 491.0 | 905.0 | 1.0 | 14.25 | 40.5 | 134.75 | 684.0 | 1.0 | 1.00 | 1.0 | 1.00 | 11.0 |

Download table as TSV
